# Structural modeling, mutation analysis, and *in vitro* expression of usherin, a major protein in inherited retinal degeneration and hearing loss

**DOI:** 10.1016/j.csbj.2020.05.025

**Published:** 2020-06-10

**Authors:** Dongmei Yu, Junhuang Zou, Qian Chen, Tian Zhu, Ruifang Sui, Jun Yang

**Affiliations:** a Department of Ophthalmology and Visual Sciences, Moran Eye Center, University of Utah, Salt Lake City, UT, United States; b Department of Ophthalmology, Peking Union Medical College Hospital, Peking Union Medical College, Chinese Academy of Medical Sciences, Beijing, China; c Department of Neurobiology and Anatomy, University of Utah, Salt Lake City, UT, United States; d Division of Otolaryngology, Department of Surgery, University of Utah, Salt Lake City, UT, United States

**Keywords:** DCC, deleted in colorectal cancer, FN3, fibronectin III, GMQE, global quality estimation score, hFc, human Fc fragment, HGMD, Human Gene Mutation Database, I-TASSER, Iterative Threading ASSEmbly Refinement, LE, laminin EGF, LG, laminin globular, LGL, laminin globular-like, LN, laminin N-terminal, mFc, mouse Fc fragment, NCBI, National Center for Biotechnology Information, QMEAN, qualitative model energy analysis score, QSQE, Quaternary Structure Quality Estimation, RMSD, root mean square deviation, RP, retinitis pigmentosa, SMTL, SWISS-MODEL template library, TM-score, template modeling score, USH, Usher syndrome, Protein folding, Recombinant protein expression, Photoreceptor, Hair cell, Structural model, Cell adhesion, Membrane protein, Usher syndrome, Retinitis pigmentosa

## Abstract

Usherin is the most common causative protein associated with autosomal recessive retinitis pigmentosa (RP) and Usher syndrome (USH), which are characterized by retinal degeneration alone and in combination with hearing loss, respectively. Usherin is essential for photoreceptor survival and hair cell bundle integrity. However, the molecular mechanism underlying usherin function in normal and disease conditions is unclear. In this study, we investigated structural models of usherin domains and localization of usherin pathogenic small in-frame mutations, mainly homozygous missense mutations. We found that usherin fibronectin III (FN3) domains and most laminin-related domains have a β-sandwich structure. Some FN3 domains are predicted to interact with each other and with laminin-related domains. The usherin protein may bend at some FN3 linker regions. RP- and USH-associated small in-frame mutations are differentially located in usherin domains. Most of them are located at the periphery of β-sandwiches, with some at the interface between interacting domains. The usherin laminin epidermal growth factor repeats adopt a rod-shaped structure, which is maintained by disulfide bonds. Most missense mutations and deletion of exon 13 in this region disrupt the disulfide bonds and may affect local protein folding. Despite low expression of the recombinant entire protein and protein fragments in mammalian cell culture, usherin FN3 fragments are more robustly expressed and secreted than its laminin-related fragments. Our findings provide new insights into the usherin structure and the disease mechanisms caused by pathogenic small in-frame mutations, which will help inform future experimental research on diagnosis, disease mechanisms, and therapeutic approaches.

## Graphical abstract

**Figure:**
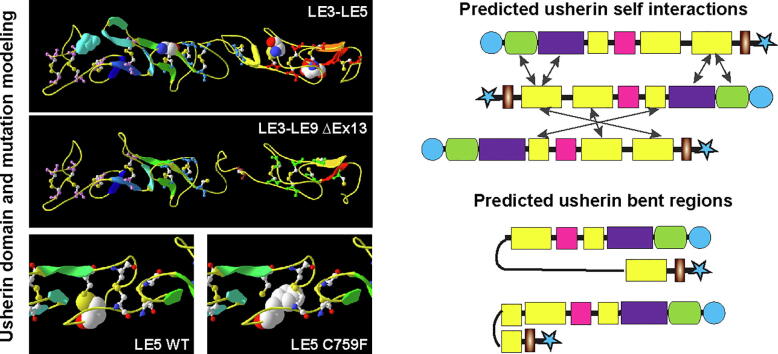

## 1 Introduction

Mutations in *USH2A* are the major cause of Usher syndrome (USH) and autosomal recessive nonsyndromic retinitis pigmentosa (RP), which account for 30–70% and ~8% of the two diseases, respectively [1], [2], [3]. While RP is a large heterogeneous group of retinal degenerative diseases, USH is characterized as RP combined with sensorineural hearing loss and is the leading cause of inherited deaf-blindness in the world. All of these diseases are incurable. Currently, more than 1,500 *USH2A* gene variants have been identified and curated in the Human Gene Mutation Database (HGMD), the clinically significant human genetic variant database ClinVar, and the Leiden Open Variation Database (LOVD)-USHbases. It has been demonstrated that a combination of two truncating *USH2A* mutations cause more severe vision and hearing impairments [4], [5]. However, nearly 700 of the identified *USH2A* gene variants are rare small in-frame variants, whose pathogenicity is usually uncertain. For example, missense variant C759F had long been thought to be pathogenic and the most common RP mutation until a recent report showing the absence of retinal degeneration in two healthy siblings carrying homozygous C759F variant [6]. Since then, the pathogenicity of the C759F variant has been debated [7]. To investigate the pathogenicity of *USH2A* small in-frame variants, several groups have localized the variants along the *USH2A* gene [2], [8], [9], [10], [11], but no obvious correlation of these variants with patient phenotypes has been identified. Furthermore, for the small group of known pathogenic *USH2A* missense variants, it remains unclear how these variants cause diseases.

**Fig. 1:**
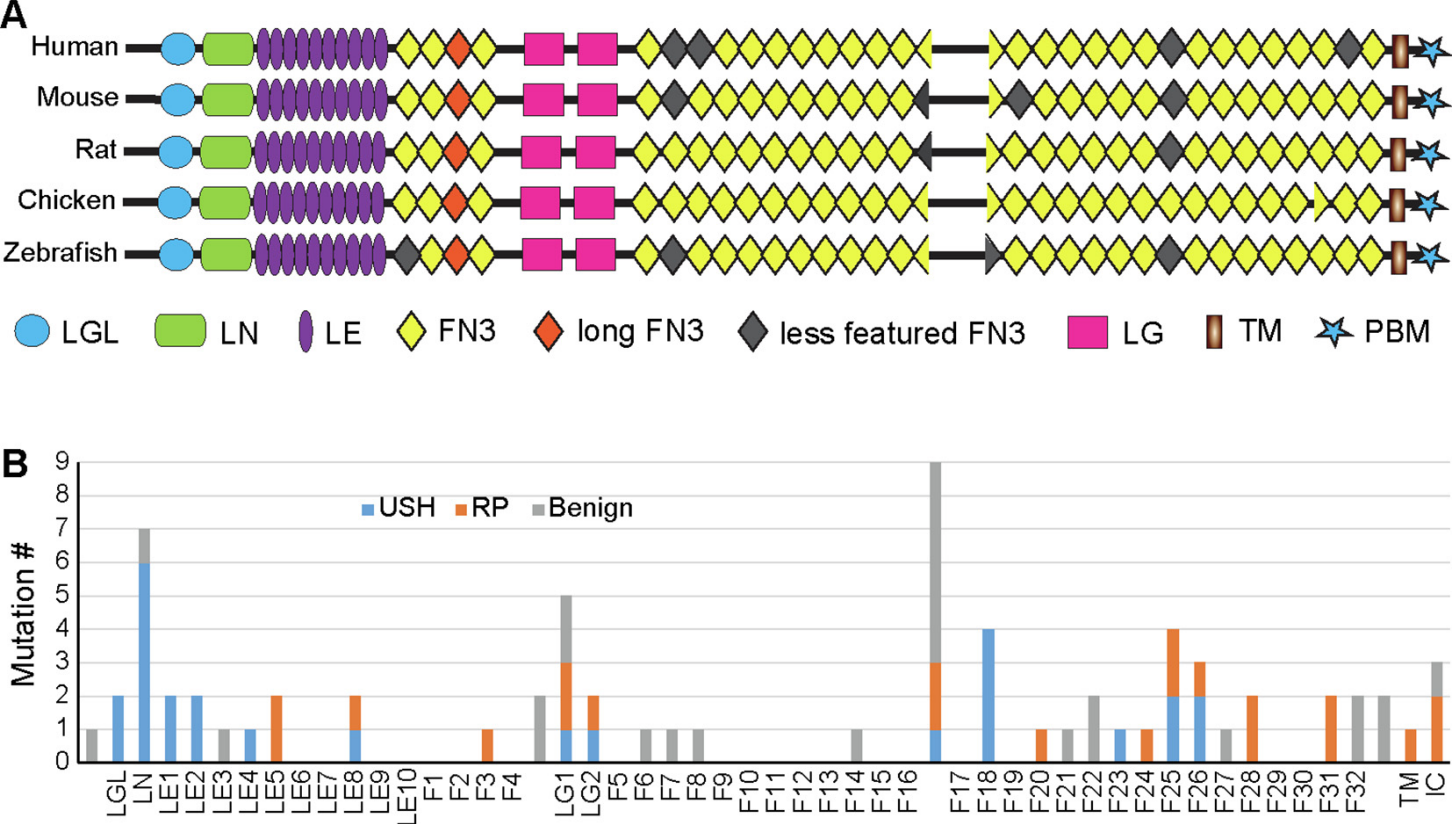
USH- and RP-associated pathogenic homozygous missense mutations tend to be located at the usherin N- and C-terminal regions, respectively. (A) Alignment of usherin domains across different species. Less featured FN3 domains are not annotated in the NCBI usherin RefSeq records. Long FN3 domains have a long CD loop (see Figs. 3A and 4A). (B) Distribution of USH- and RP-associated pathogenic and benign homozygous missense variants in various usherin domains. TM, transmembrane domain; IC, intracellular region.

Usherin, the protein product of the *USH2A* gene, is a single-pass transmembrane protein and has 5,202 amino acids (aa) in humans (Fig. 1A). The ectodomain of usherin occupies ~97% of the protein and has been seldom studied. This ectodomain contains 1 laminin globular-like (LGL), 1 laminin N-terminal (LN), 10 laminin epidermal growth factor (LE), 2 laminin globular (LG), and 32 fibronectin III (FN3) domains. Among them, the LE region has been shown to interact with fibronectin [12] and collagen [13] *in vitro*. The usherin intracellular C-terminal end has a PDZ-binding motif (PBM), which interacts with other USH and deafness proteins, such as whirlin and PDZD7, in retinal photoreceptors and inner ear hair cells [14], [15], [16], [17]. Mouse genetic studies have shown that usherin is essential for photoreceptor survival and hair cell stereociliary bundle integrity [18], [19], [20], [21], [22], [23]. Recent studies in zebrafish models have further found that loss of *ush2a* expression induces photopigment mislocalization, abnormal formation of lysosome-like structures, and elevated autophagy levels [24], [25], [26]. However, the exact molecular mechanism of usherin function in healthy and diseased photoreceptors and hair cells remains to be elucidated.

The most common *USH2A* mutations for USH (c.2299delG) [27], [28], [29] and RP (p.C759F, though still debatable) [30], [31] are located in exon 13, which is 642 base pairs long and in frame. It has been hypothesized that skipping exon 13 has a therapeutic potential, because the majority of the usherin protein can be produced theoretically, except for a fragment between LE4 and LE8. A phase I/II clinical trial based on this strategy is currently undertaken aiming to treat retinal degeneration. According to the recent interim analysis (press release on the ProQR website, March 31, 2020), 2 of 8 treated patients showed encouraging evidence of efficacy. The exon 13-skipping strategy has also been studied in an *Ush2a*^ΔEx12^ mouse model, where the mouse counterpart of human usherin exon 13 deletion (ΔEx13) protein, usherin ΔEx12, was localized normally in photoreceptors and hair cells [32], [33]. In mice, usherin ΔE12 protein is able to fully function in cochlear hair cells [32], [33], while its function in photoreceptors has not been clearly demonstrated, because the *Ush2a* null mice as a baseline control have a very weak retinal degeneration phenotype [18]. Despite these promising findings from clinical trial and mouse studies, it is unknown whether and how the usherin ΔEx13 protein behaves similarly to its wild-type counterpart in photoreceptors and hair cells.

Charactering the usherin three-dimensional (3D) atomic structure is essential to understand the molecular mechanism of usherin function and is also valuable to address translational questions regarding the pathogenicity of *USH2A* small in-frame variants, the *USH2A* genotype-phenotype correlation, and the development of therapeutic strategies. Currently, to solve the usherin structure is technically unfeasible by X-ray crystallography, nuclear magnetic resonance, or single particle cryo-electron microscopy, because of usherin protein’s large size, membrane residence, and potential flexible conformations. The structures of individual usherin domains have also not been solved. Fortunately, most usherin domains belong to families of domains that have been extensively studied in other proteins. Therefore, the structures of these domains, which are homologous to usherin domains, can be identified as templates for modeling. For the usherin domains whose templates are unavailable, current computational advances allow structural modeling using combined sequence/structure-based threading and *ab initio* modeling.

In this study, we applied structural modeling to investigate usherin domain structures and interactions and analyze the locations of *USH2A* RP- and USH-associated homozygous mutations and their effects on usherin structure. We also investigated the potential effect on usherin structure of the Exon 13-skipping therapeutic strategy. Finally, we explored the feasibility of producing usherin and its fragments in mammalian cultured cells. Our findings lay out a valuable foundation for future experimental determination of usherin structure and function. The structural models built in this study provide novel insights into the usherin mechanism of action and the pathogenic consequences of *USH2A* small in-frame mutations. Furthermore, our study on the structural effect of exon 13 skipping suggests that structural modeling is a potential tool for usherin therapeutic studies.

## 2 Materials and methods

### 2.1 *USH2A* missense mutation analysis

*USH2A* missense variants and small in-frame insertions and deletions (INDELs, < 20 amino acids) were searched in the publically accessible version of the Human Gene Mutation Database (HGMD, http://www.hgmd.cf.ac.uk/ac/newgenes.php, RRID: SCR_001888), the clinically significant human genetic variant database ClinVar (https://www.ncbi.nlm.nih.gov/clinvar/, RRID: SCR_006169), and the Leiden Open Variation Database (LOVD)-USHbases (https://databases.lovd.nl/shared/genes/USH2A, RRID: SCR_006566). From this search, 436, 65, and 343 pathogenic or likely pathogenic missense and small in-frame INDEL variants were found, respectively. To determine the pathogenicity of these variants and their association with USH and RP, 119 original literature reports in the MEDLINE literature database (RRID: SCR_002185) were scrutinized. The pathogenic variants identified as homozygous and the sole genetic changes in patients were included in our studies. In addition, four pathogenic *USH2A* missense mutations that met the same criteria and had not been curated in the HGMD, ClinVar database, or LOVD-USHbases were identified in our own 284 USH and RP patients [34]. These patients were recruited in the Ophthalmic Genetics Clinic at the Peking Union Medical College Hospital, Beijing, China.

### 2.2 DNA plasmid and antibody generation

Five usherin cDNA fragments (NP_067383: 1-562 aa, 543-1838 aa, 1832-2800 aa, 2792-3839 aa, and 3833-5194 aa) were generated from mouse retinal total RNA by reverse transcription and polymerase chain reaction (RT-PCR) using TRIzol reagent and ThermoScript™ RT-PCR system (Life Technologies). The five usherin cDNA fragments were then partially digested and ligated sequentially to generate full-length usherin cDNA, which was inserted into pcDNA3.1(-) plasmid (Life Technologies). After confirmation by DNA sequencing, the full-length usherin cDNA was found to have a missense variant c.12442G > A (p.D4148N) at the F24-F25 linker region. The usherin ectodomain (1-4924 aa, NP_067383) construct was generated from the full-length construct by removing the cDNA encoding the usherin transmembrane and intracellular region (4925-5193 aa) and adding a C-terminal in-frame Strep tag. Other usherin fragments used in this study were amplified from the usherin full-length construct by PCR and then inserted into their destination vectors. Usherin LN-LE10 (310-1035 aa), F19-F21 (3582-3854 aa), and F11-F32 (2521-4923 aa) fragments were cloned into pSec-Tag2A-mFc-Biotin vector [35]. Usherin F19-F32 fragment (3582-4918 aa) was cloned into pDisplay™ vector (Life Technologies). Usherin F15-F18 (2903-3570 aa) and F17-F21 (3428-3854 aa) fragments were cloned into pET11-his-PP vector (gift from Christopher Hill, University of Utah). Usherin F5-F15 (1947-2993 aa), F17-F32 (3441-4923 aa), F17-F21 (3441-3854 aa), F17-F23 (3441-4053 aa), F25-F32 (4153-4923 aa), F1-LG2 (1055-1857 aa), and F1-F15 (1055-2993 aa) fragments were cloned into pCEP-Pu vectors with various tags [36].

The procedure to generate usherin A3 antibody was similar to what was described previously [37]. Briefly, the cDNA encoding the mouse usherin fragment from 445 to 790 aa was cloned into pET28 vector and was expressed in BL21-CodonPlus (DE3)-RIPL cells (Agilent Technologies, Santa Clara, CA, USA). The usherin recombinant fragment was then purified from the bacterial cell lysate using Ni^2+^-charged His•Bind resin (EMD Millipore, Billerica, MA, USA) under denaturing conditions and used to immunize a rabbit. The same usherin fragment was cross-linked with agarose resin and used to affinity purify the antibody from the obtained rabbit serum.

### 2.3 Bioinformatic analyses

Pairwise sequence comparison of human usherin full-length protein and individual FN3 domains with their counterparts in different species was conducted using the BLASTp suite at the National Center for Biotechnology Information (NCBI) website (RRID: SCR_001010). Sequence alignment of the 32 human usherin FN3 domains was performed using the PROMALS3D multiple sequence and structure alignment server (http://prodata.swmed.edu/promals3d/promals3d.php, RRID: SCR_018161). These two tasks were conducted using default parameters. The obtained human FN3 domain sequence alignment data was applied to generate an unrooted maximum likelihood phylogenetic tree using the Molecular Evolutionary Genetics Analysis program (MEGA X, RRID: SCR_000667) [38], [39]. The WAG substitution model, gamma distributed rates among sites, and 95% site coverage cutoff for partial deletion of gaps/missing data treatment were chosen, as suggested by the feature of Find Best DNA/Protein Models in the MEGA X program. The phylogenetic tree was viewed using FigTree v1.4.4 program (RRID: SCR_008515).

Homology modeling of human usherin individual domains and homo- and heteromeric domain complexes was conducted using the automated mode with default settings by the ProMod3 modeling engine at the SWISS-MODEL server (https://swissmodel.expasy.org/, RRID: SCR_018123) [40], [41], [42]. When multiple models of the usherin LE and FN3 domains were built by SWISS-MODEL, representative models were chosen based on their high sequence identity, large fragment coverage, high scores of Global Model Quality Estimation (GMQE), Qualitative Model Energy ANalysis (QMEAN), and Quaternary Structure Quality Estimation (QSQE, dimer models only). For the human usherin F16-F17 linker and intracellular fragment (NP_996816, 3088–3448 aa and 5064-5202aa, respectively), whose models were unable to be built by SWISS-MODEL because of lack of templates, the Iterative Threading ASSEmbly Refinement (I-TASSER) server (https://zhanglab.ccmb.med.umich.edu/I-TASSER/, RRID: SCR_014627) was utilized to build the models using default settings [43]. Swiss-PdbViewer 4.1.0 (RRID: SCR_013295) was used to display and analyze the built usherin models.

### 2.4 Protein expression in mammalian and bacterial cells

HEK293 (ATCC, CRL1573) and COS-7 (ATCC, CRL1651) cells were grown in Dulbecco’s modified Eagle’s medium (DMEM) supplemented with 10% (v/v) fetal bovine serum, 100 units/ml penicillin, and 100 μg/ml streptomycin (ThermoFisher). HEK293-EBNA cells (ATCC, CRL-10852) were maintained in DMEM F12 medium supplemented with 10% (v/v) fetal bovine serum, 100 units/ml penicillin, 100 μg/ml streptomycin, and 250 μg/ml Geneticin (ThermoFisher). FreeStyle™ 293-F and Expi293F™ cells were cultured according to the manufacturer’s instructions (ThermoFisher).

Lipofectamine 2000 reagent (ThermoFisher) was used to transfect HEK293, COS-7, HEK293-EBNA, and FreeStyle™ 293-F cells with usherin cDNA plasmids. The transfection of Expi293F™ cells was conducted using the Expi293 Expression System (ThermoFisher). The transfected HEK293-EBNA cells were enriched by culturing in the maintenance medium supplemented with 1 μg/ml puromycin (ThermoFisher). During usherin protein expression, HEK293-EBNA cells were grown in DMEM F12 medium supplemented with 100 units/ml penicillin and 100 μg/ml streptomycin. All other transfection and subsequent protein expression procedures followed the manufacturer’s instructions. Protein expression was examined in these cell lines at 2–6 days post-transfection.

To express usherin proteins in bacterial cells, usherin cDNA plasmids were chemically transformed into BL21-CodonPlus (DE3)-RIPL cells (Agilent Technologies, Santa Clara, CA, USA). Usherin protein expression was induced by adding 1 mM IPTG into the BL21 cell culture, which underwent a linear growth phase. Protein expression was examined 3 h after induction.

### 2.5 Protein preparation, SDS-polyacrylamide gel electrophoresis (PAGE), immunoblotting, and immunostaining

Mammalian cultured cells and media were separated by centrifugation twice at a speed of 250 × g–5,000 × g for 10–15 min, depending on the cell type. The obtained culture media were analyzed either directly or after concentration by acetone precipitation at −20 °C overnight. The obtained mammalian cells were lysed in lysis buffer (50 mM Tris-HCl pH 7.5, 150 mM NaCl, 0.5% Triton X-100, 5 mM EDTA, 1 X protease inhibitor, and 1 mM DTT) at 4 °C for 20 min and cleared by centrifugation at 18,000 × g for 10 min. BL21-CodonPlus (DE3)-RIPL cells were separated from culture medium by centrifugation at 13,500 × g for 10 min. To solubilize usherin full-length protein from cell membranes, transfected FreeStyle™ 293-F cells were sonicated in lysis buffer with 0.5% CHAPS, 1% NP-40, or 1% Triton X-100 for 5 s 3 times, gently rocked for 1 h, and centrifuged at 18,000 × g for 20 min. The resulting supernatants and pellets were collected. His-, hFc-, and mFc-tagged proteins were purified from either culture media or mammalian cell lysates using HisPur™ Ni-NTA resin (ThermoFisher) and Protein G Sepharose™ (Fisher Scientific), respectively. The culture media, mammalian cell lysates, bacterial cell lysates, or purified proteins were added with SDS-PAGE loading buffer (6X, 375 mM Tris pH 6.8, 12% SDS, 60% glycerol, 600 mM DTT, and 0.06% bromophenol blue) and incubated at 42 °C or boiled at 100 °C for 10 min. SDS-PAGE and immunoblotting procedures were the same as described previously [44]. Polyclonal rabbit anti-Strep-tag II antibody (Abcam, ab76949, RRID: AB_1524455, 1:1000), monoclonal mouse anti-FLAG M2 antibody (Sigma-Aldrich, F1804, RRID: AB_262044, 1:1000), horseradish peroxidase conjugated goat anti-mouse antibody (Jackson ImmunoResearch, 115-035-146, RRID: AB_2307392, 1:10000), horseradish peroxidase conjugated goat anti-rabbit antibody (Jackson ImmunoResearch, 111-035-144, RRID: AB_2307392, 1:10000), and horseradish peroxidase conjugated donkey anti-human antibody (Jackson ImmunoResearch, 709-035-149, RRID: AB_2340495, 1:10000) were used for immunoblotting analyses.

Immunostaining was conducted using the protocol previously described [44]. Briefly, transfected COS-7 cells were fixed in a mixture of methanol and acetone (1:1) at −20 °C for 10 min. The cells were then double stained using a polyclonal rabbit antibody against Na^+^/K^+^-ATPase α (ATP1A1) (Santa Cruz, sc-28800, RRID: AB_2290063, 1:500) and a monoclonal mouse antibody against HA (Sigma-Aldrich, H3663, RRID: AB_262051, 1:500).

## 3 Results

### 3.1 Pathogenic homozygous small in-frame mutations are unevenly distributed in usherin domains

Usherin domain arrangement has been well conserved during evolution (Fig. 1A). However, in the NCBI conserved domain database, some usherin FN3 domains are not annotated consistently among different species (Fig. 1A and Table S1), despite their highly conserved sequence (Table S2). This is likely due to these domains having less typical FN3 features. To avoid the confusion when we refer to FN3 domains in different species in this paper, we re-annotated FN3 domains according to their chicken counterparts (Table S1). To understand the functional importance of the various usherin domains, we analyzed the distribution of *USH2A* pathogenic mutations along the protein. Because nonsense and frameshift mutations usually eliminate the expression of entire gene products, and the mutant alleles in compound heterozygous status and other mutations in the same patients may have confounding effects, we focused on *USH2A* missense mutations and small in-frame insertion and deletion (INDEL, <20 amino acids) mutations that were homozygous and the only genetic changes in patients.

The *USH2A* gene has 436, 65, and 343 pathogenic or likely pathogenic missense and small in-frame INDEL variants listed in the public version of HGMD, the ClinVar database, and the LOVD-USHbases, respectively, although many of these variants were redundant in the three databases. After examining the information in the databases and reading 119 original literature reports, we identified 42 *USH2A* pathogenic missense mutations and 1 *USH2A* small in-frame duplication mutation that met our selection criteria (Table 1). Additionally, we screened the mutations that had been identified from our own 284 *USH2A* patients [34] and found 4 more *USH2A* pathogenic missense mutations that met our selection criteria (Table 1). Among all these selected pathogenic homozygous mutations (hereafter the word homozygous will be omitted for simplicity when our selected mutations are mentioned), 26 were associated with USH and 21 were associated with RP. Interestingly, mutation C934W (c.2802 T > G) was found in both USH and RP patients. Mutations of R4192 to histidine (R4192H, c.12575G > A) and cysteine (R4192C, c.12574C > T) were found in USH and RP, respectively. Furthermore, we included 25 *USH2A* benign homozygous missense variants from the LOVD-USHbases as negative controls (Table 2). While these benign missense variants were distributed quite evenly along the entire usherin protein, the missense mutations associated with USH were highly enriched in the usherin N-terminal LGL, LN, LE1, LE2, and LE4 domains, and the missense and in-frame duplication mutations associated with RP were enriched in the C-terminal FN3-28 (F28) domain, F31 domain, transmembrane domain, and intracellular region (Fig. 1B). The LN, LG1, F18, F25, and F26 domains were highly enriched with pathogenic missense mutations, while 5 of the 10 LE domains and 23 of the 32 FN3 domains were free of these mutations. The linker region between F16 and F17 (F16-F17 linker) was conserved across species and contained 3 pathogenic missense mutations (Fig. 1A and Table S2). In summary, *USH2A* pathogenic small in-frame mutations are enriched in most laminin-related domains, specific FN3 domains, F16-F17 linker, transmembrane domain, and intracellular region, suggesting that these usherin regions are functionally important.

**Table 1:** *USH2A* pathogenic small in-frame mutations investigated in this study.^1^

Mutations	cDNA changes	Domain	Phenotype	PubMed #
p.G268R	c.802G > A	LGL	USH	29490346 [54]
p.T281K	c.842C > A	LGL	USH	22135276 [55]
p.C319Y	c.956G > A	LN	USH	10729113 [29]
p.R334W	c.1000C > T	LN	USH	10738000 [56]
				18452394 [57]
				26338283 [58]
p.N346H	c.1036A > C	LN	USH	21174530 [59]
				27318125 [4]
p.D347H	c.1039G > C	LN	USH	26927203 [5]
p.C419F	c.1256G > T	LN	USH	22135276 [55]
				15241801 [60]
				27318125 [4]
p.G516V	c.1547G > T	LN	USH	21738395 [61]
p.C520R	c.1558 T > C	LE1	USH	25333064 [62]
p.C536R	c.1606 T > C	LE1	USH	27957503 [63]
				26927203 [5]
p.C620Y	c.1859G > A	LE2	USH	24944099 [2]
p.C638F	c.1913G > T	LE2	USH	24944099 [2]
p.G713R	c.2137G > C	LE4	USH	21738395 [61]
p.C759F	c.2276G > T	LE5	RP	10775529 [64]
				14970843 [30]
				21151602 [65]
				12525556 [66]
				28041643 [67]
				23591405 [68]
				25649381 [10]
				26927203 [5]
				28894305 [69]
p.D778Y	c.2332G > T	LE5	RP	25649381[10]
p.C934W	c.2802 T > G	LE8	USH & RP	26338283 [58]
				this report
p.P1242S	c.3724C > T	F3	RP	25649381 [10]
p.G1526R	c.4576G > A	LG1	RP	25356976 [70]
p.G1671D	c.5012G > A	LG1	RP	26667666 [71]
p.L1673P	c.5018 T > C	LG1	USH	27318125 [4]
p.G1734R	c.5200G > C	LG2	RP	20309401 [72]
p.G1840V	c.5519G > T	LG2	USH	29490346 [54]
p.L3145F	c.9433C > T	F16-F17 linker	RP	26806561 [73]
p.C3267R	c.9799 T > C	F16-F17 linker	USH	19683999 [74]
p.C3358Y	c.10073G > A	F16-F17 linker	RP	28894305 [69]
p.W3521R	c.10561 T > C	F18	USH	27318125 [4]
p.L3536R	c.10607 T > G	F18	USH	24944099 [2]
p.G3546R	c.10636G > A	F18	USH	22004887 [9]
				24944099 [2]
				24516651 [75]
p.T3571M	c.10712C > T	F18	USH	25575603 [76]
				19683999 [74]
				28894305 [69]
p.R3719H	c.11156G > A	F20	RP	this report
p.P4035L	c.12104C > T	F23	USH	this report
p.L4148P	c.12443 T > C	F24	RP	27596865 [77]
p.W4175G	c.12523 T > G	F25	RP	26352687 [78]
p.R4192H	c.12575G > A	F25	USH	2213276 [55]
p.R4192C	c.12574C > T	F25	RP	30718709 [79]
p.T4234P	c.12700A > C	F25	USH	25575603 [76]
p.P4269R	c.12806C > G	F26	USH	20507924 [11]
p.N4292D	c.12874A > G	F26	RP	25133751 [80]
p.Y4331C	c.12992A > G	F26	USH	26927203 [5]
p.G4489D	c.13466G > A	F28	RP	25324289 [81]
p.T4498_T4500dup	c.13491_13499dupTACTCTCAC	F28	RP	28894305 [69]
p.S4748F	c.14243C > T	F31	RP	25324289 [81]
p.G4763R	c.14287G > C	F31	RP	25133613 [82]
p.S5060P	c.15178 T > C	TM	RP	this report
p.P5078R	c.15233C > G	IC	RP	25324289 [81]
p.V5145I	c.15433G > A	IC	RP	30718709 [79]

1 The *USH2A* pathogenic small in-frame mutations in this table were identified in homozygosity and as the only genetic mutations in patients. The phenotypes are from the patients whose mutations are in homozygosity.

**Table 2:** *USH2A* benign missense variants investigated in this study^1^.

Mutations	cDNA changes	Domain	PubMed #
p.A125T	c.373G > A		24831256 [83]
			17405132 [84]
			19683999 [74]
			24944099 [2]
			20052763 [85]
p.E478D	c.1434G > C	LN	22004887 [9]
p.D644V	c.1931A > T	LE3	17405132 [84]
			24944099 [2]
			22009552 [86]
p.R1486G	c.4456A > G		17085681 [8]
			19683999 [74]
			12112664 [87]
p.R1486K	c.4457G > A		24831256 [83]
			17405132 [84]
			1968999 [74]
			2494099 [2]
			20052763 [85]
p.L1572F	c.4714C > T	LG1	22135276 [55]
			17405132 [84]
			24944099 [2]
p.I1665T	c.4994 T > C	LG1	17405132 [84]
			1708681 [8]
			24944099 [2]
			20507924 [11]
			22004887 [9]
p.T2106I	c.6317C > T	F6	17405132 [84]
			19683999 [74]
			24944099 [2]
			12112664 [87]
			20513143 [88]
			22009552 [86]
			20052763 [85]
			20507924 [11]
			22004887 [9]
p.I2169T	c.6506 T > C	F7	24831256 [83]
			17405132 [84]
			19683999 [74]
			12112664 [87]
			20052763 [85]
p.R2292H	c.6875G > A	F8	24944099 [2]
p.L2886F	c.8656C > T	F14	22135276 [55]
			24944099 [2]
p.N3099S	c.9296A > G	F16-F17 linker	24944099 [2]
p.T3115A	c.9343A > G	F16-F17 linker	22135276 [55]
			24944099 [2]
p.D3144N	c.9430G > A	F16-F17 linker	24944099 [2]
p.N3199D	c.9595A > G	F16-F17 linker	22135276 [55]
			24944099 [2]
p.E3411A	c.10232A > C	F16-F17 linker	17405132 [84]
			19683999 [74]
			24944099 [2]
			20052763 [85]
p.E3411D	c.10233A > C	F16-F17 linker	12112664 [87]
p.T3835I	c.11504C > T	F21	17405132 [84]
			24944099 [2]
			20052763 [85]
			22004887 [9]
			20507924 [11]
			24498627 [89]
p.M3868V	c.11602A > G	F22	17405132 [84]
			12112664 [87]
			24944099 [2]
			20052763 [85]
			22004887 [9]
			20507924 [11]
p.P3893T	c.11677C > A	F22	20507924 [11]
p.V4433L	c.13297G > T	F27	22135276 [55]
p.G4838E	c.14513G > A	F32	22135276 [55]
p.R4848Q	c.14543G > A	F32	22135276 [55]
p.K5026E	c.15076A > G		22135276 [55]
p.R5031W	c.15091C > T		22135276 [55]
			20507924 [11]
p.S5188G	c.15562A > G	IC	22135276 [55]

1 The *USH2A* benign missense variants in this table were homozygous benign missense variants collected from the LOVD-USHbases.

### 3.2 Most pathogenic homozygous missense mutations are located in loops of the usherin laminin-related domain models except those in the LE model

**Fig. 2:**
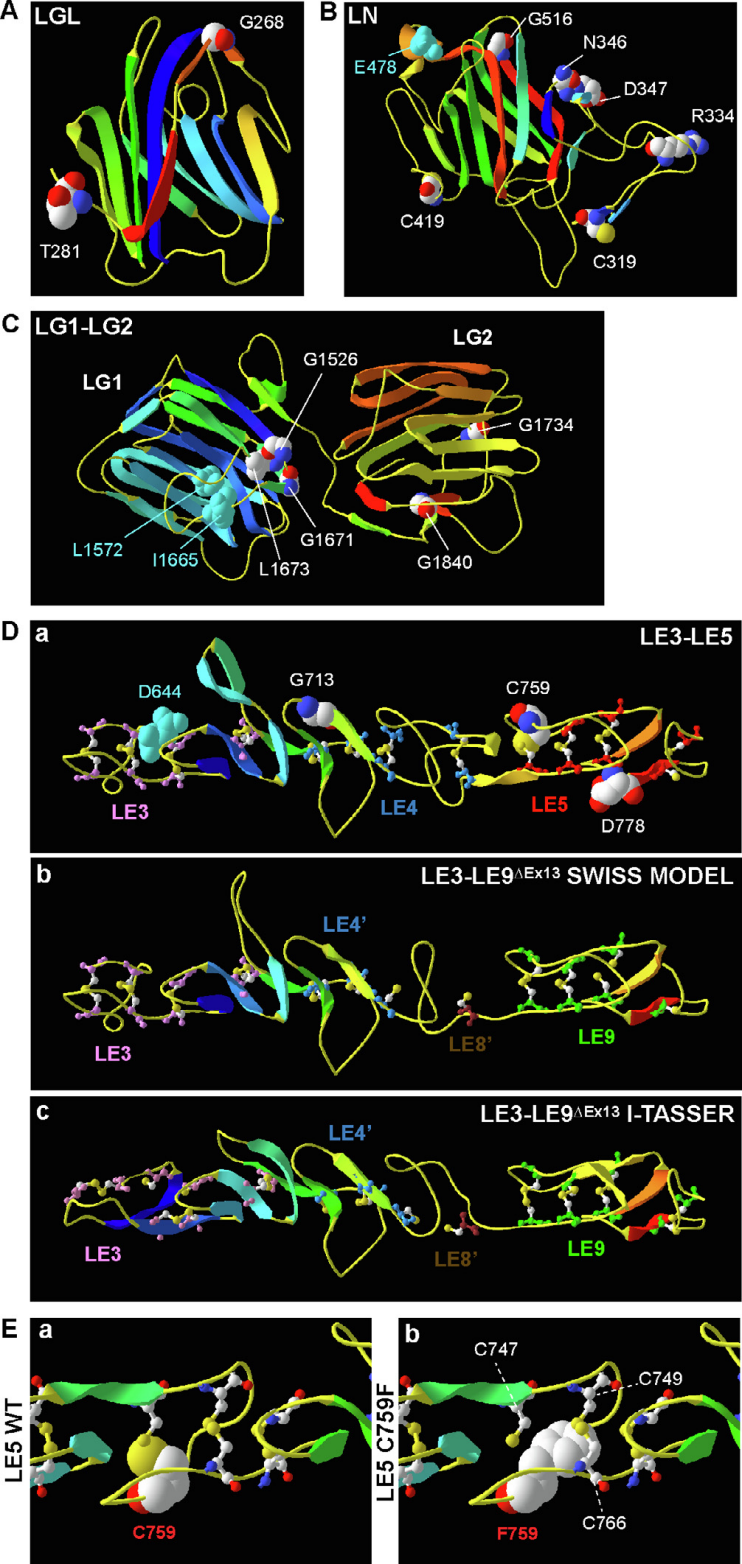
Pathogenic homozygous missense mutations are mostly located at the periphery of usherin laminin-related domain models, except the LE domain models. (A–C) Pathogenic homozygous missense mutations are located at the periphery of the β-sandwich in usherin LGL (A), LN (B), and LG (C) domain models. (D) Usherin LE repeat model shows a rod-shaped structure, which is maintained by four disulfide bonds per LE domain, as exemplified by LE3-LE5 (a). Skipping of exon 13 in human *USH2A* gene deletes a protein fragment from LE4 to LE8. The cysteines in the remaining LE4 (LE4′) and LE8 (LE8′) domains cannot form the four disulfide bonds as in an intact LE domain as predicted by homology modeling (b) and I-TASSER (c). (E) C759 forms a disulfide bond with C747 in usherin LE5 domain (a). The most common RP-associated mutation C759F affects this disulfide bond and may sterically affect the disulfide bond between C749 and C766 (b). The structural models in this figure are shown in a ribbon model and colored with a rainbow scheme for secondary structures (Blue: N-terminal end, Red: C-terminal end). Wild-type residues, where pathogenic missense mutations (CPK coloring) and benign missense variants (Cyan) occur, are shown in a space-filling model. Cysteines that form disulfide bonds in D and E are shown in a ball-stick model. CPK coloring for atoms: white, carbon; red, oxygen; blue, nitrogen; and yellow, sulfur. (For interpretation of the references to color in this figure legend, the reader is referred to the web version of this article.)

To investigate the impact of *USH2A* pathogenic small in-frame mutations on protein structure and potentially function, we built structural models of usherin domains. Each usherin laminin-related domain had at least one homologous template found in the SWISS-MODEL template library (SMTL). We chose the representative templates 5hp6.1.A (extracellular arabinanase), 4plm.1.A (netrin-1), 1npe.1.B (laminin gamma-1 chain), and 6cw1.1.A (neurexin-1) to build the LGL, LN, LE, and LG models, respectively (Fig. 2 and Table 3). These templates showed 22–30% sequence identity and covered more than 91% of the usherin laminin-related domains (Table 3). The global quality estimation score (GMQE) and the qualitative model energy analysis score (QMEAN) of the models were in the range of 0.53 to 0.66 and −5.47 to −4.42, respectively (Table 3).

**Table 3:** Templates and model quality scores from homology modeling of human usherin fragments and complexes.

Fragment(s)	Range (aa)	Template ID	Template description	Seq identity	Coverage	GMQE	QMEAN	QSQE
LGL	146–283	5hp6.1.A	Extracellular arabinanase	22.2%	91%	0.53	−5.43	N/A
LN	295–516	4plm.1.A	Netrin-1	26.0%	92%	0.62	−5.47	N/A
LE3-LE5	641–795	1npe.1.B	Laminin gamma-1 chain	30.4%	95%	0.66	−4.82	N/A
LE3-LE9 ΔEx13	641–722, 938–1000	1npe.1.B	Laminin gamma-1 chain	28.6%	92%	0.62	−4.42	N/A
LG1-LG2	1519–1868	6cw1.1.A	Neurexin-1	23.7%	96%	0.64	−4.44	N/A
F3	1242–1357	2ic2.2.A	CG9211-PA	20.2%	81%	0.51	−3.96	N/A
F18	3503–3586	2djs.1.A	Ephrin type-B receptor 1	28.4%	96%	0.64	−2.52	N/A
F20	3680–3767	1wk0.1.A	FN3-containing protein 3A	29.3%	93%	0.63	−2.88	N/A
F28	4444–4516	4pln.1.C	Neogenin	26.4%	99%	0.65	−2.45	N/A
F31	4732–4809	3e0g.1.A	Leukemia inhibitory factor receptor	22.7%	96%	0.54	−7.44	N/A
F23-F26	3964–4351	6mfa.1.A	Fibronectin F4-F7	19.2%	88%	0.53	−5.16	N/A
		1fnf.1.A	Fibronectin F7-F10	16.5%	89%	0.50	−4.43	N/A
F25-F26	4161–4351	4m4r.1.A	Ephrin type-A receptor 4	23.1%	97%	0.63	−4.05	N/A
F20-F23 and F20-F23	3680–4057	3t1w.1.A	Fibronectin F7FbF8F9	18.5%	90%	0.56	−3.98	0.12
F20-F23 and F25-F28	3680–4057 + 4161–4516	3bpn.1	Interleukin 4 receptor α chain + Interleukin 13 receptor α1 chain	11.2%	60%	0.29	−6.15	0.21
F18 and F25-F26	3503–3586 + 4161–4351	5x83.1	Netrin receptor DCC	21.5%	90%	0.56	−3.25	0.10
TM	5041–5070	2 k21.1.A	Potassium voltage-gated channel subfamily E member	25.0%	93%	0.63	−0.82	N/A
LN-LE3 and F18	295–691 + 3503–3586	4pln.1	Neogenin F4-F5 + Netrin-1	30.3%	91%	0.65	−3.51	0.33
LN-LE3 and F25-F26	295–691 + 4161–4351	4pln.1 (model 1)	Neogenin F4-F5 + Netrin-1	27.4%	92%	0.64	−4.55	0.27
		4plo.1 (model 2)	Netrin receptor DCC F4-F5 + Netrin-1	28.0%	91%	0.63	−4.27	0.39
		4urt.1 (model 3)	Netrin receptor DCC F5-F6 + Netrin-1	28.0%	92%	0.64	−3.97	0.34

**Fig. 3:**
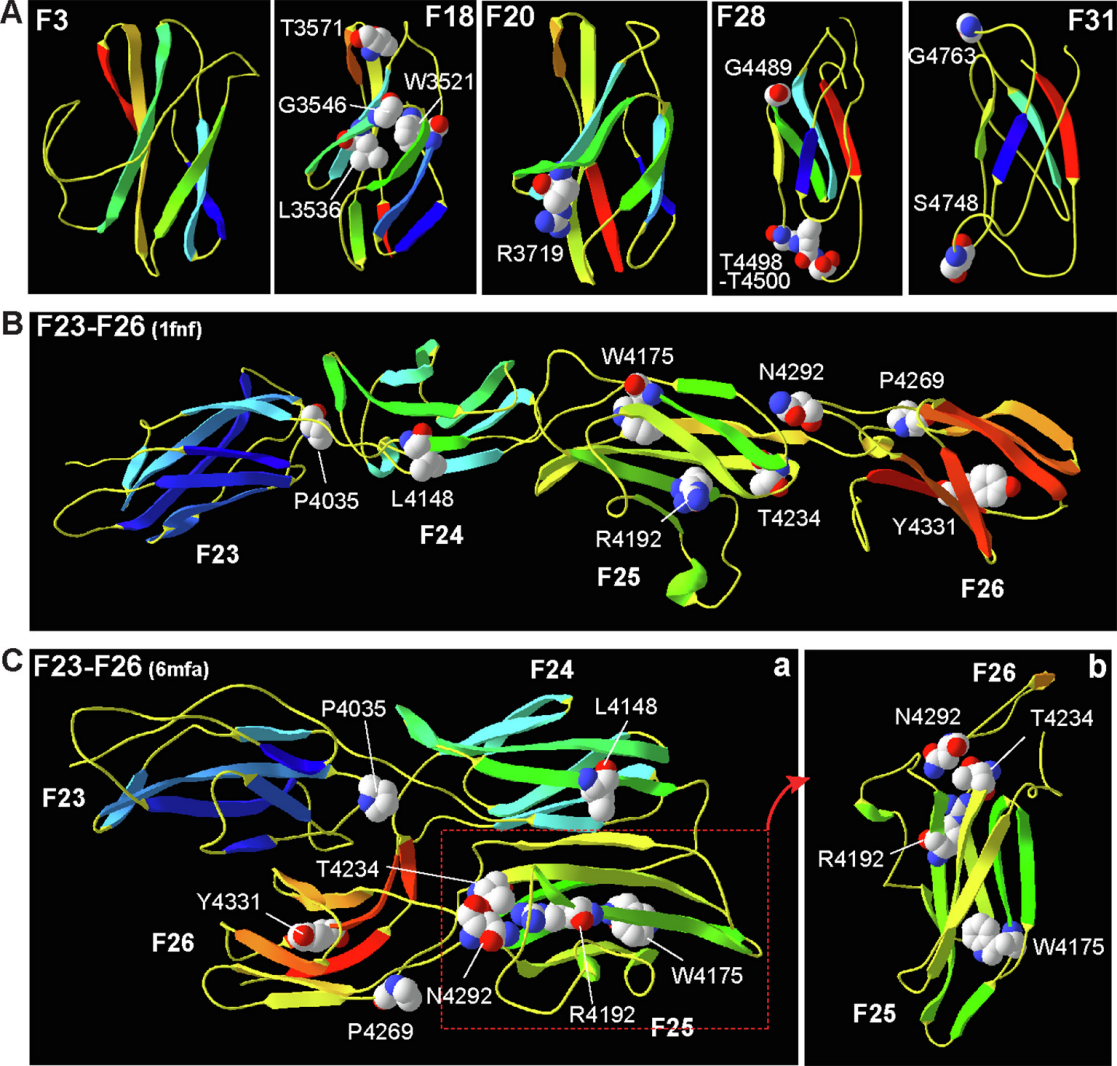
Most pathogenic homozygous small in-frame mutations are absent at the core β-sandwich of usherin FN3 domains. (A) W3521R, G3546R, T3571M, R3719H, G4489D, T4498_T4500dup, S4748F, and G4763R mutations are located at the two poles of usherin F18, F20, F28, and F31 models, while L3536R is located on strand C in usherin F18 model. Note that the F3 model started at residue 6 and P1242S, which is at the first residue in the F3 domain, was not shown. (B) A linear usherin F23-F26 model based on template 1fnf shows that P4035L, L4148P, W4175G, P4269R, and N4292D mutations are located in loops or β-strand ends, while R4192H/C, T4234P, and Y4331C mutations are located on β-strands in the β-sandwiches. (C) A folded usherin F23-F26 model based on template 6mfa shows that the usherin F23-F26 fragment bends at the F24-F25 linker (a). The positions of most missense mutations in this model are similar to those in the linear model, except L4148P and T4234P, which are located on strand G in F24 and at the N-terminal end of strand F in F25, respectively. Interestingly, T4234 in F25 and N4292 in loop BC of F26 are located within a distance of 4Å (b). Panel b shows the model region highlighted in red in panel a. The model presentation and atom color scheme are the same as in Fig. 2. (For interpretation of the references to color in this figure legend, the reader is referred to the web version of this article.)

The LGL model had a β-sandwich topology with strands β1, β6, β7, β8, and β11 on one sheet and strands β2, β3, β4, β5, and β9 on the other sheet (Fig. 2A). G268 was located in the middle of strand β10 on the top of the β-sandwich. T281 was positioned in a loop following strand β11. The LN model had a β-sandwich topology with strands β1, β5, β7, β8, β9, and β11 on one sheet and strands β4, β6, β10, and β12 on the other sheet (Fig. 2B). A β-strand hairpin (β2 and β3) and three small α-helixes were located outside the β-sandwich. R334, N346, D347, C419, and G516 were located in the loops at the model periphery. C319 was in the middle of the β2 strand. The combined LG1 and LG2 model showed that each of the LG domains had a β-sandwich topology (Fig. 2C). The relative spatial orientation of the LG1 and LG2 β-sandwiches varied depending on templates used. In the model built on template 6cw1.1.A, the LG1 had a β-sandwich topology of strands β1, β3, β4, β5, β6, β11, and β15 on one β-sheet and strands β2, β7, β8, β9, β13, and β14 on the other β-sheet. Strands β10, β12, and a small α-helix were present at the periphery of the β-sandwich. G1526 was in the loop preceding strand β1, and G1671 and L1673 were located on strand β12. The LG2 had a β-sandwich topology of strands β1, β3, β8, β9, β10, and β12 on one β-sheet, and strands β2, β4, β5, β6, β7, and β11 on the other β-sheet. G1734 was located in the loop between strands β2 and β3, while G1840 was located on strand β11. As a validation control experiment, we also examined whether three known benign missense variants (Table 2) affect the above models. E478D was on a peripheral α-helix in the LN model (Fig. 3B), and I1665T was in a peripheral loop between strands β11 and β12 in the LG1 model (Fig. 3C). However, L1572F was on strand β4 in the LG1 β-sandwich core (Fig. 3C). Further analysis found that the side chains of L and F residues were similar in size and thus the L1572F variant was not expected to alter the β-sandwich structures. In summary, the pathogenic missense mutations in the LGL, LN, and LG domains are mainly located outside their jelly-roll topology β-sandwiches and unlikely to disrupt the core structure of the domains.

The usherin LE3-LE5 repeat model was built based on the laminin γ-1 template 1npe.1.B (Fig. 2D), because no homologous template was available for the entire LE1-LE10 region in the SMTL. The LE3-LE5 model had a straight rod-like shape. The folding of each LE domain was maintained by 4 disulfide bonds between cysteine 1 and cysteine 3, cysteine 2 and cysteine 4, cysteine 5 and cysteine 6, and cysteine 7 and cysteine 8. These disulfide bonds separated each LE domain into loops a, b, c, and d. Some loops accommodated antiparallel β-strands. In LE5, C759 was cysteine 3 and formed a disulfide bond with C747 (cysteine 1). The mutation C759F was predicted to break the disulfide bond and might sterically affect the formation of the disulfide bond between cysteine 2 and cysteine 4, i.e., C749 and C766 (Fig. 2Da and E). In fact, in the usherin LE1-LE10 region, the majority of the pathogenic missense mutations occurred at cysteines (Table 1), which would break the disulfide bonds and affect the local LE folding in the LE1-LE10 rod structure. G713 and D778 were positioned at loop b in LE4 and loop d in LE5, respectively, and both were at the surface of the model (Fig. 2Da). Furthermore, benign missense variant D644V (Table 2) was also localized at the surface of the model at loop a in LE3 (Fig. 2Da).

To investigate the effect of exon 13 skipping on the LE repeat structure, we built a model of LE3-LE9 without exon 13 (LE3-LE9^ΔEx13^) based on the same template used for the LE3-LE5 model. The LE3-LE9^ΔEx13^ model (Fig. 2Db) showed that the remaining halves of LE4 and LE8 domains were unable to fold as an intact LE domain. However, cysteines 1 to 4 in the remaining LE4 half were able to form disulfide bonds and maintain the relatively normal structural folding. The two adjacent LE3 and LE9 domains also appeared normal. To further confirm the LE3-LE9^ΔEx13^ model generated by homology modeling, we utilized the I-TASSER program, which created protein structure models by sequence-based and structure-based threading and *ab initio* modeling [43]. The top LE3-LE9^ΔEx13^ model generated from this approach had a C-score of −0.07, an estimated template modeling score (TM-score) of 0.70 ± 0.12, and an estimated root mean square deviation (RMSD) of 4.8 ± 3.1Å, which were relatively high confidence scores [45]. The folding defect of the truncated LE4 and LE8 in the I-TASSER model (Fig. 2Dc) was similar to that in the homology model (Fig. 2Db). Consistently, the structures adjacent to the deletion region, the remaining LE4 part, and LE9, appeared relatively normal (Fig. 2Dc).

### 3.3 Most pathogenic homozygous small in-frame mutations are located at the periphery of usherin FN3 models, with some at the conserved residues between adjacent FN3 domains

A typical FN3 domain has a β-sandwich topology of strands A, B, and E on one β sheet and strands C, D, F, and G on the other. The loops between the β-strands on the two poles of the β-sandwich are highly variable and usually involved in interactions with partners [46]. In the SMTL, templates were found to build models for up to four consecutive usherin FN3 domains. Using these templates, we built models for individual FN3 domains, F3, F18, F20, F28, and F31, and the 4 consecutive FN3 domains, F23-F26, where pathogenic small in-frame mutations were found.

**Fig. 4:**
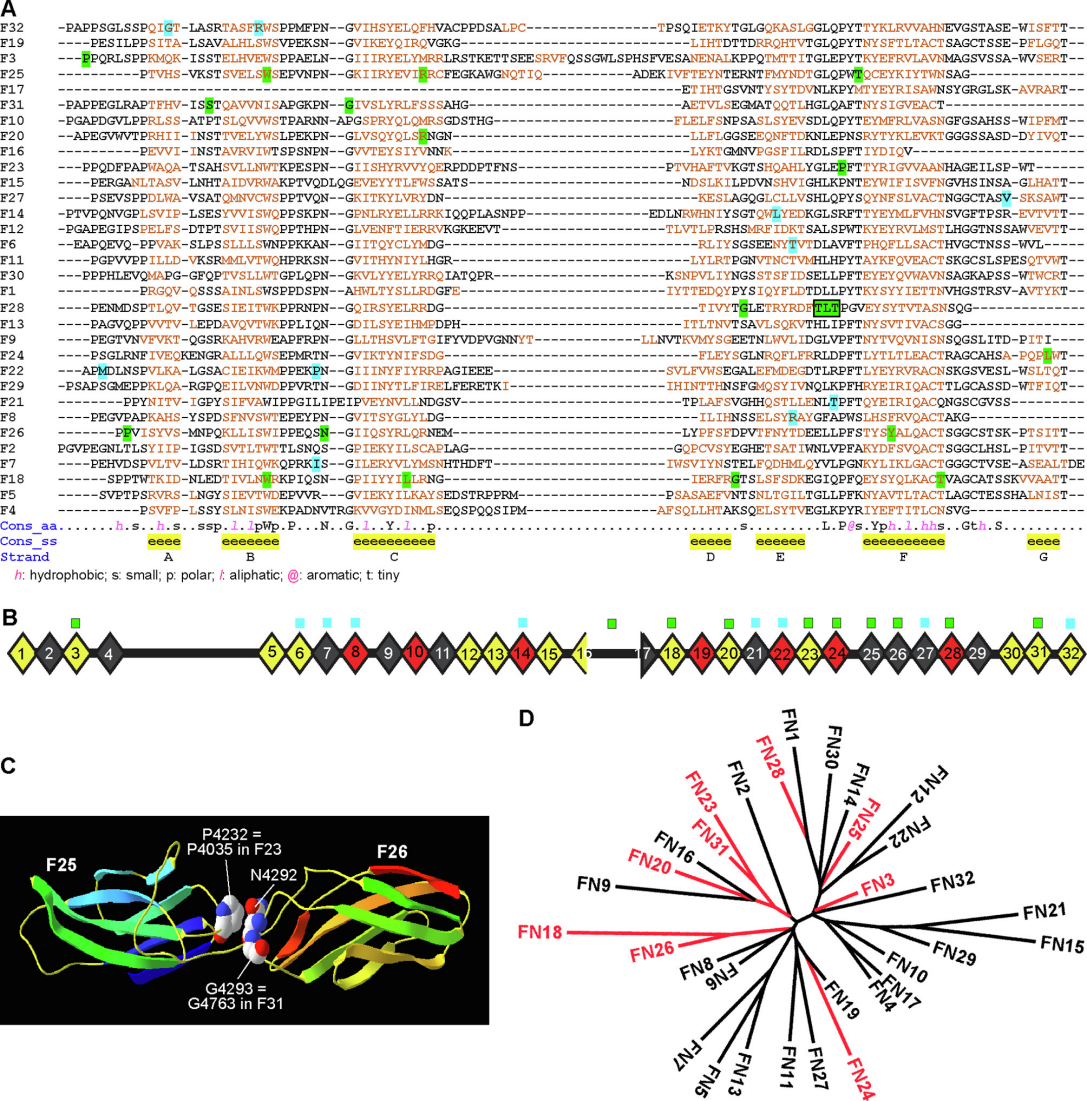
Pathogenic homozygous small in-frame mutations occur at conserved usherin FN3 residues and preferentially in sequence-related usherin FN3 domains. (A) Sequence alignment of human usherin FN3 domains. Red residues were predicted to form β-strands, as indicated in the Cons_ss and Strand lines at the bottom. Highly and intermediately conservative residues are indicated in line Cons_aa by uppercase letters and other symbols, respectively. The key to other symbols is shown below. Pathogenic missense mutations highlighted in green, but not benign missense variants highlighted in cyan, occur in all types of conserved residues. (B) Both pathogenic missense mutations (green squares) and benign missense variants (cyan squares) are distributed in all types of evolutionally conserved FN3 domains. The pathogenic missense mutations are enriched in usherin F18-F31 region. The more (red) and less (grey) evolutionally conserved FN3 domains are defined by their sequence identity across other species higher and lower than that of the entire protein, respectively (Table S2). (C) A ribbon model of usherin F25-F26 fragment shows that P4232 in loop EF of F25 is within a distance of 4Å with N4292 and G4293 in loop BC of F26. P4232 and G4293 are equivalent to residues P4035 in F23 and G4763 in F31, respectively, where pathogenic missense mutations occur. The model presentation and atom color scheme are the same as in Fig. 2. (D) Phylogenetic tree of human usherin FN3 domains shows that the FN3 domains hosting pathogenic missense mutations (red) tend to be in the same small clades. (For interpretation of the references to color in this figure legend, the reader is referred to the web version of this article.)

The templates for the F3, F18, F20, F28, and F31 domains were 2ic2.2.A (CG9211-PA), 2djs.1.A (ephrin type B receptor 1), 1wk0.1.A (FN3 domain-containing protein 3A), 4pln.1.C (neogenin), and 3e0g.1.A (leukemia inhibitory factor receptor), respectively. These templates shared a sequence identity of 20.2–29.3% with the usherin FN3 domains and covered 81–99% of these domains (Table 3). The F18 and F20 models showed a standard FN3 β-sandwich topology, although strands F and G of F18 and strand G of F20 had a short break in the middle (Fig. 3A). The F3, F28, and F31 models adopted an atypical FN3 β-sandwich topology. The presumed strand D in F3 and F31 and the presumed strand A in F28 and F31 did not form a β-strand (Fig. 3A). The strand G sequence in F28 and F31 was missing (Figs. 3A and 4A). In addition, the F3 model had a long loop CD (Figs. 3A and 4A). The P1242S mutation in the F3 domain was located at the first residue and was absent in the built model, which started at residue 6. Thus, this mutation was outside of any β-strands in the core β-sandwich, which was verified by the sequence alignment in Fig. 4A. In the F18 model, L3536 was located in the middle of strand C, and W3521, G3546, and T3571 were located at the C-terminal end of strands B, D, and F, respectively (Fig. 3A). R3719 in the F20 model was located at the C-terminal end of strand C, and G4489 in the F28 model was present at the C-terminal end of strand D (Fig. 3A). The T4498_T4500 duplication in the F28 model and S4748 and G4763 in the F31 model were in loop EF, loop AB, and loop CD, respectively (Fig. 3A). No benign homozygous small in-frame variants were found in these FN3 domains (Table 2).

Two fibronectin fragments F7-F10 (1fnf.1.A) and F4-F7 (6mfa.1.A) in the SMTL had a similar sequence identity (16.5% vs. 19.2%) and coverage range (89% vs. 88%) with the usherin F23-F26 fragment (Table 3). The models generated from these two templates also had similar quality scores (GMQE: 0.50 vs. 0.53 and QMEAN: −4.43 vs. −5.16), suggesting that the usherin F23-F26 fragment may adopt two conformations. The model based on template 1fnf.1.A showed that the usherin F23-F26 fragment had an extended linear conformation (Fig. 3B). Strands G in the F23, F24, and F25 domains were broken into 2 small β-strands, while strand G in F26 was missing. Several α-helices existed at loops in F24, F25, and F26. P4035 was positioned in loop EF of F23. L4148 was positioned at the N-terminal end of the second strand G in F24. W4175 was at the C-terminal end of strand B, and R4192 and T4234 were on strand C and strand F, respectively, in F25. P4269 and N4292 were in the loops and Y4331 was on strand F in F26 (Fig. 3B). Again, no benign homozygous small in-frame variants were found in usherin F22-F26 region (Table 2).

The usherin F23-F26 model based on template 6mfa.1.A showed a bent conformation (Fig. 3C). Hydrogen bonds were present along the interfaces between F23 and F26, between F23 and F24, and between F23 and F25 (not shown). Strands G in F23, F24, and F26 and strand B in F25 were broken into 2 small β-strands. The presumed strand A in F23 adapted a loop conformation. A small α-helix existed in F25 (Fig. 3C). The positions of P4035, W4175, R4192, P4269, N4292, and Y4331 in this bent model were similar to those in the extended linear model (Fig. 3B and C). However, L4148 was changed to the middle of strand G in F24, and T4234 was changed to the N-terminal end of strand F in F25. Interestingly, the van der Waals surfaces of T4234 in F25 and N4292 in F26 were next to each other, within a distance of 4Å (Fig. 3Cb), indicating that the two residues might participate in the association between F25 and F26 in the bent conformation.

We next investigated whether the pathogenic small in-frame mutations occurred at special residues in usherin FN3 domains. Sequence alignment of the 32 human usherin FN3 domains showed that, while 21 of them had the typical 7 β-strands, F4, F6, F8, F13, F21, F23, F25, F28, and F31 had no strand G; F16 and F17 were partial FN3 domains; and F3 had a long CD loop (Fig. 4A). Among the 32 usherin FN3 domains, the highly conserved residues included a tryptophan in strand B; a proline, asparagine, and glycine in loop BC; a tyrosine in strands C and F; a leucine and proline in loop EF; and a glycine and serine in loop FG (Fig. 4A). While the benign missense variants (highlighted in cyan in Fig. 4A, Table 2) were not located at any of these conserved residues, the pathogenic small in-frame mutations (highlighted in green in Fig. 4A) occurred at 5 of these highly conserved residues, 6 intermediately conserved residues, and 6 nonconserved residues, indicating that the pathogenic mutations occur at either a common or a unique position of the FN3 domains. Three of the 5 highly conserved residues, P4035, N4292, and G4763, were within a distance of 4Å of neighboring FN3 domains, as exemplified in Fig. 3Cb for N4292 and by an independent homology model of usherin F25-F26, where the corresponding residues were P4232 in F25 and N4292 and G4293 in F26 (Fig. 4C). We concluded that the three missense mutations at the conserved residues likely affect the association between adjacent FN3 domains.

As described above, when compared with human sequences, all usherin FN3 domains were evolutionally conserved with sequence identities of 57.1–87.1% in mice, 56.5–84.0% in rats, 53.0–90.3% in chickens, and 37.4–69.4% in zebrafish (Table S2). The FN3 domains with higher and lower sequence identities than those of the entire proteins were distributed randomly in the FN3 repeats (Fig. 4B). Both the pathogenic and benign small in-frame variants were located in all types of evolutionarily conserved FN3 domains, although the pathogenic small in-frame variants were highly enriched in the F17-F32 repeats (Fig. 4B).

To further reveal the sequence similarity among the 32 human usherin FN3 domains, phylogenetic analysis was performed. The FN3 domains that hosted pathogenic small in-frame mutations had a weak tendency to be clustered together (Fig. 4D). For example, F18 and F26 were exclusively in a small clade, and F20, F23, and F31 were in the same clade with two other FN3 domains. It is generally believed that protein domains with similar sequences tend to have similar biological functions or interacting partners. Therefore, the usherin pathogenic small in-frame mutations are likely to affect the FN3 domains with similar functions or interacting partners.

### 3.4 Usherin FN3 repeats are predicted to interact with themselves, which may be affected by pathogenic missense mutations

**Fig. 5:**
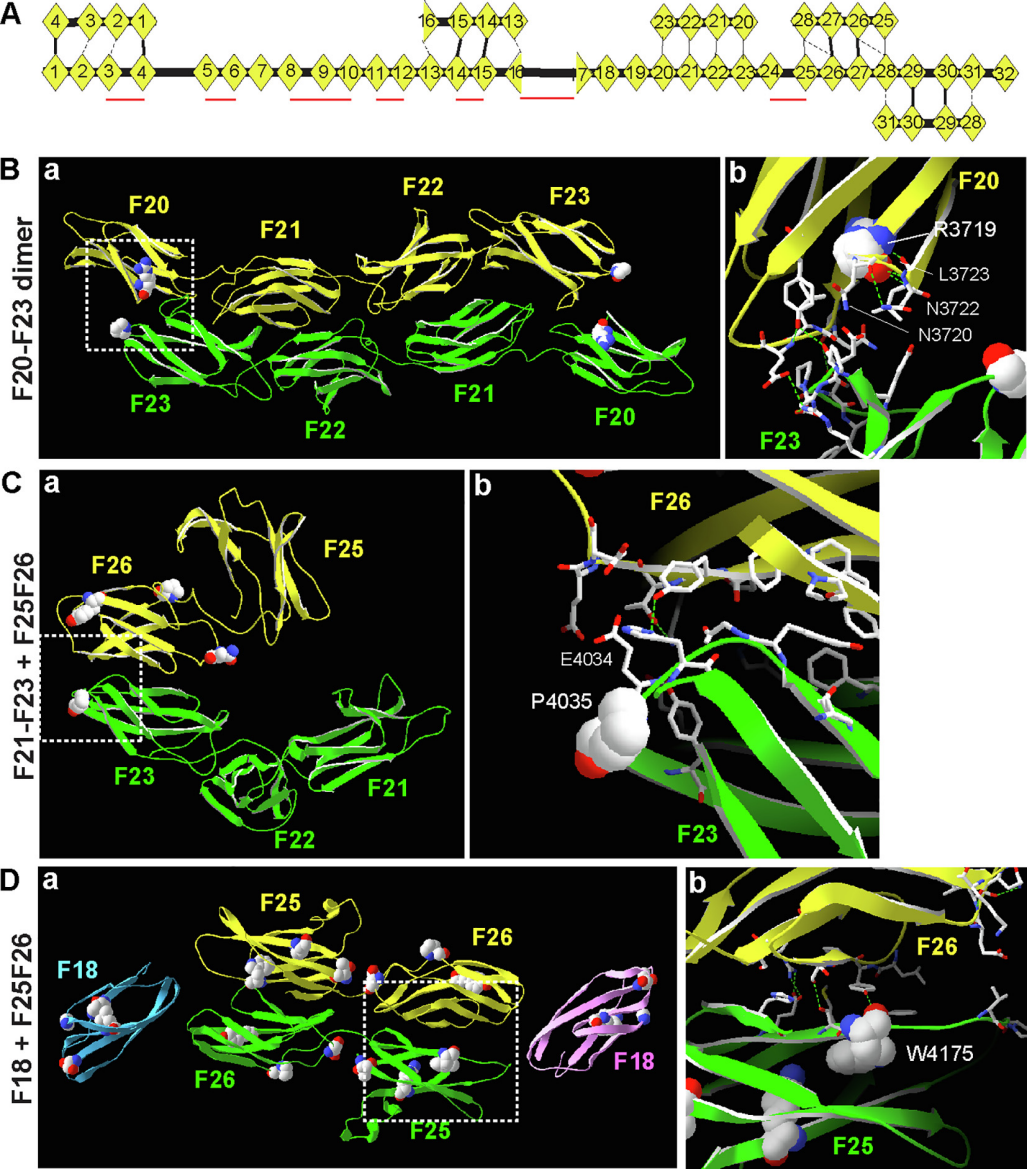
Some usherin FN3 domains are predicted to interact with each other. (A) A scheme showing usherin FN3 regions that can form antiparallel homodimers, as predicted by homology modeling. Thick, thin, and dashed lines between FN3 domains in the dimers indicate decreasing numbers of residues at the interface. Red bars indicate the FN3 linkers with a conserved residue length. (B) An antiparallel homodimer model of usherin F20-F23 fragment (a). N3720, N3722, and L3723 are at the interface between F20 and F23 domains. R3719 in F20 is localized immediately next to N3720 and forms hydrogen bonds (green dashed lines) with N3722 and L3723 (b). (C) Usherin F21-F23 and F25-F26 fragments are predicted to form a heterodimer through an association between F23 and F26 domains (a). P4035 in F23 is immediately next to the interface residue E4034 (b). (D) A 2:2 heterodimer model of usherin F18 and F25-F26 fragments, where two F25-F26 fragments are antiparallel and F18 is at the two ends of the F25-F26 fragments (a). W4175 in F25 is an interface residue with F26 (b). The models are presented as ribbons and colored differently for protein fragments. The residues at the interface of protein fragments are shown in a ball-stick model. Wild-type residues that are altered in pathogenic missense mutations are shown in a space-filling model. The regions within the white frame in the a panels are enlarged and shown in the b panels. (For interpretation of the references to color in this figure legend, the reader is referred to the web version of this article.)

Homology modeling found head-to-tail homodimer models for usherin F1-F4, F13-F16, F20-F23, F25-F28, and F28-F31 fragments, but not for any other four consecutive usherin FN3 domains (Fig. 5A). The models with the highest GMQE score for these FN3 fragments were consistently built on template 3t1w.1.A (oncofetal fibronectin F7FbF8F9 domains, Table S3). In these homodimer models, hydrogen bonds were formed and interface residues (defined hereafter as residues within a 4-Å distance) were found between different FN3 fragments (Fig. 5A). These FN3 homodimer models suggest that usherin may interact at FN3 domains intermolecularly and intramolecularly. In the F20-F23 model (Fig. 5Ba and Table 3), R3719 in F20 was localized next to N3720 and formed hydrogen bonds with N3722 and L3723 (Fig. 5Bb). N3720, N3722, and L3723 were at the interface between F20 and F23. Therefore, mutation R3719H may affect the association between F20 and F23.

Homology modeling was also conducted on several pairs of different four consecutive usherin FN3 domains. All examined pairs showed a similar heterodimer model based on the same template 3bpn.1, which was a complex between interleukin-4 receptor α chain and interleukin-13 receptor α1 chain. In an example model (Fig. 5C and Table 3), a heterodimer of the mutation-enriched F20-F23 and F25-28 fragments was formed through an interaction between F23 and F26 (Fig. 5Ca). P4035 in F23 was next to interface residue E4034 (Fig. 5Cb), suggesting that the P4035L mutation may affect the interaction of F23 with F26.

To further investigate the potential interactions between different usherin FN3 domains, we focused on usherin F18 and F25-F26 fragments, where the pathogenic missense mutations were the most enriched (Fig. 1A). A heterodimer model with a 2:2 stoichiometry was built based on the template of the netrin receptor deleted in colorectal cancer (DCC), 5x83.1 (Table 3). In this model, two F25-F26 fragments formed an antiparallel dimer, and two F18 domains associated with F25 and F26 at the two ends of the dimer (Fig. 5Da). W4175 in F25, which was also a highly conserved residue among all 32 FN3 domains (Fig. 4A), was an interface residue with F26 (Fig. 5Db). Thus, the W4175G mutation may directly disrupt the interactions between F25 and F26.

Notably, P4035L in F23 was predicted to affect the interaction of F23 with F26 in the F21-F23 and F25-F26 heterodimer model (Fig. 5C), but was not at the interface between F23 and F20 in the F20-F23 homodimer model (Fig. 5B). F23 was also predicted to interact with F24, F25, and F26 simultaneously in the bent F23-F26 monomer model (Fig. 3C). Furthermore, F26 and F28 were predicted to associate with two other FN3 domains simultaneously in the F25-F28 homodimer model (Fig. 5A and Table S3). All of these models suggested that three and four FN3 domains were able to interact directly. We therefore tested whether the three usherin fragments of F1-F4, F18, and F23-F26 or the three usherin fragments of F18, F25, and F31, where the pathogenic missense mutations were located, were able to bind among one another directly and form a ternary complex. No model was able to be built for these combinations of usherin FN3 fragments using the SWISS-MODEL program, suggesting that these FN3 fragments were unable to form a ternary complex.

The various models of usherin FN3 domains built in this study (Fig. 3, Fig. 5) suggested that the usherin FN3 repeat region might be able to adopt functionally relevant linear and folded conformations. In this case, the length of linkers between adjacent FN3 domains might determine the capability and tightness of the folded conformation. We thus examined the conservation of usherin FN3 linker length across different species. The F16-F17 linker was consistently approximately 360 residues long (Fig. 5A and Table 4), sufficient for a loose folding of the usherin FN3 repeat region. The linker lengths of F3-F4, F8-F9, and F24-F25 were evolutionarily conserved and approximately 10–15 residues long (Fig. 5A and Table 4), which could allow a tight folding between two adjacent FN3 domains, such as F24 and F25 in the model shown in Fig. 3C. Additionally, the linker lengths were less conserved in the F17-F32 region than in the F5-F16 region (Fig. 5A), suggesting that the F17-F32 region, where the pathogenic missense mutations are enriched, may have a less conserved conformation than the F5-F16 region.

**Table 4:** Usherin FN3 linker length (aa) in different species.

Linker position	Human NP_996816	Mouse NP_067383	Rat NP_001289148	Chicken XP_015139380	Zebrafish XP_009291422
F1-F2	1	24	24	13	19
F2-F3	3	3	3	3	10
F3-F4	10	10	10	8	10
F5-F6	2	2	2	2	2
F6-F7	7	7	7	4	8
F7-F8	2	1	7	4	4
F8-F9	15	15	15	15	10
F9-F10	3	3	3	2	3
F10-F11	4	7	11	14	2
F11-F12	1	1	1	1	1
F12-F13	4	4	4	1	9
F13-F14	18	1	21	1	22
F14-F15	4	4	4	4	6
F15-F16	10	23	10	10	17
F16-F17	361	362	362	358	374
F17-F18	8	10	2	8	22
F18-F19	3	3	19	3	3
F19-F20	3	3	7	9	16
F20-F21	9	15	13	2	16
F21-F22	9	12	12	19	20
F22-F23	3	3	3	10	24
F23-F24	8	4	4	4	4
F24-F25	10	10	10	10	10
F25-F26	20	20	9	15	3
F26-F27	4	4	4	4	15
F27-F28	4	3	14	3	4
F28-F29	12	1	1	1	1
F29-F30	8	25	25	29	25
F30-F31	1	1	11	1	6
F31-F32	16	16	16	27	33

Linkers with a conserved length across species are highlighted.

### 3.5 The pathogenic missense mutation in the usherin transmembrane domain is predicted to affect protein structure, while those in the intracellular region and F16-F17 linker are not

3.5

**Fig. 6:**
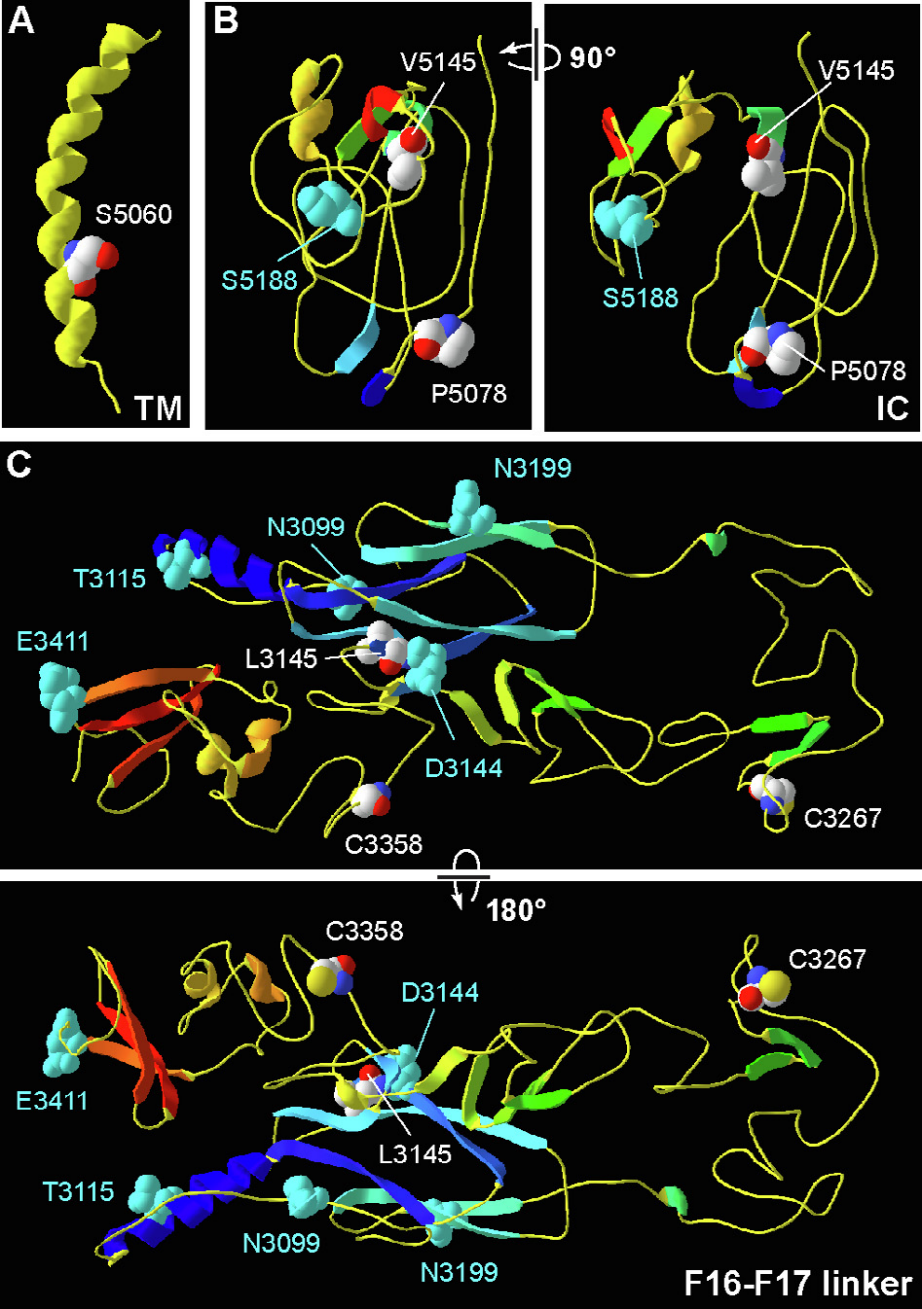
Pathogenic homozygous missense mutations are localized in the models of usherin F16-F17 linker, transmembrane domain, and intracellular region. (A) Homology modeling shows that the usherin transmembrane (TM) domain adopts a long α-helix structure. S5060P is located at the C-terminal portion of the helix. (B) The top I-TASSER model of the usherin intracellular (IC) region shows that both the pathogenic P5078R missense mutation and benign S5188G missense variant are present in a loop and the pathogenic V5145I missense mutation is at the N-terminal end of a short α-helix. The left and right panels show the same model with a 90-degree rotation. (C) The top I-TASSER model shows that the usherin F16-F17 linker is bent at a long loop region in the middle of the fragment. The pathogenic missense mutations are located at the C-terminal end of a β-strand (L3145F) and in loop regions (C3267R and C3358Y). Benign missense variants are located in a loop (N3099S), on an α-helix (T3115A) and β-strands (D3144N and N3199D), and at the C-terminal end of a β-strand (E3411A). The upper and bottom panels show the same model with a 180-degree rotation. The model presentation and atom color scheme are the same as in Fig. 2, except for the helix color in A.

Homology modeling using the 2 k21.1.A template (potassium voltage-gated channel subfamily E member) predicted that the usherin transmembrane domain formed a long α-helix (Fig. 6A and Table 3). Pathogenic mutation S5060P was located to the C-terminal portion of the α-helix, which was expected to break the α-helix structure [47]. Homology modeling however could not predict a model for the intracellular region or the usherin F16-F17 linker. We thus utilized I-TASSER to model these regions.

The top model built for the intracellular region had a C-score of −3.76, an estimated TM-score of 0.31 ± 0.10, and an estimated RMSD of 13.4 ± 4.1Å, which were relatively low confidence scores. No structural analog with a TM-score higher than 0.6 was found in the protein data bank on the Research Collaboratory for Structural Bioinformatics website, suggesting that the model was not similar to any known protein structures. In this model, the majority of the intracellular region adopted loops except four short β-strands and two short α-helixes (Fig. 6B). The pathogenic mutations P5078R and V5145I were positioned in a loop and at the N-terminal end of an α-helix, respectively (Fig. 6B). A known benign missense variant S5188G was located in a loop (Fig. 6B and Table 2). Therefore, none of the pathogenic and benign missense variants were predicted to affect the folding of the usherin intracellular region.

The top model built for the F16-F17 linker (Fig. 6C) had a better quality, with a C-score of −1.36, an estimated TM-score of 0.55 ± 0.15, and an estimated RMSD of 9.7 ± 4.6 Å. This model matched the hybrid, EGF1, EGF2, EGF3, and EGF4 domains in the crystal structure of integrin β3, 4g1eB [48], with a TM-score of 0.934. The model showed a U-shaped structure bent in the middle at a long loop region. One arm of the U-shaped model had two α-helixes and one β-sandwich, and the other arm had several antiparallel β-strands, two short α-helixes, and one 3-β-strand sheet. The pathogenic mutations, L3145F, C3267R, and C3358Y, were located at the end of a β-strand or in the middle of loops (Fig. 6C). For the known benign missense variants (Table 2), N3099S, D3441A, and E3411D were located at the end of a β-strand or in a loop, while T3115A, D3144N, and N3199D were located on an α-helix or β-strand (Fig. 6C), although these residue changes were not expected to alter the α-helix or β-strands significantly. Therefore, the pathogenic and benign missense variants may not change the structure of the F16-F17 linker.

### 3.6 FN3 domains are predicted to interact with laminin-related domains in usherin, which may be affected by pathogenic missense mutations

Netrin-1 LN and LE domains (4plm1.1A) were frequently identified as templates for the models of usherin LN and LE domains, and the FN3 domains of neogenin (4pln.1.C) and DCC (5x83.1), two netrin receptors [49], were the frequent templates for modeling usherin FN3 domains (Table 3). We thus investigated whether usherin laminin-related domains were able to bind to usherin FN3 domains by homology modeling. Preliminary results using usherin LGL-LE5 fragment with usherin F18, F1-LG2, or F25-F28 fragment generated similar models, which were complexes composed of the LN-LE3 fragment and one or two FN3 domains (not shown). To further investigate the effect of pathogenic missense mutations on the interactions between usherin LN-LE3 and FN3 domains, we performed homology modeling again using the pathogenic missense mutation-enriched LN-LE3, F18, and F25-F26 fragments.

**Fig. 7:**
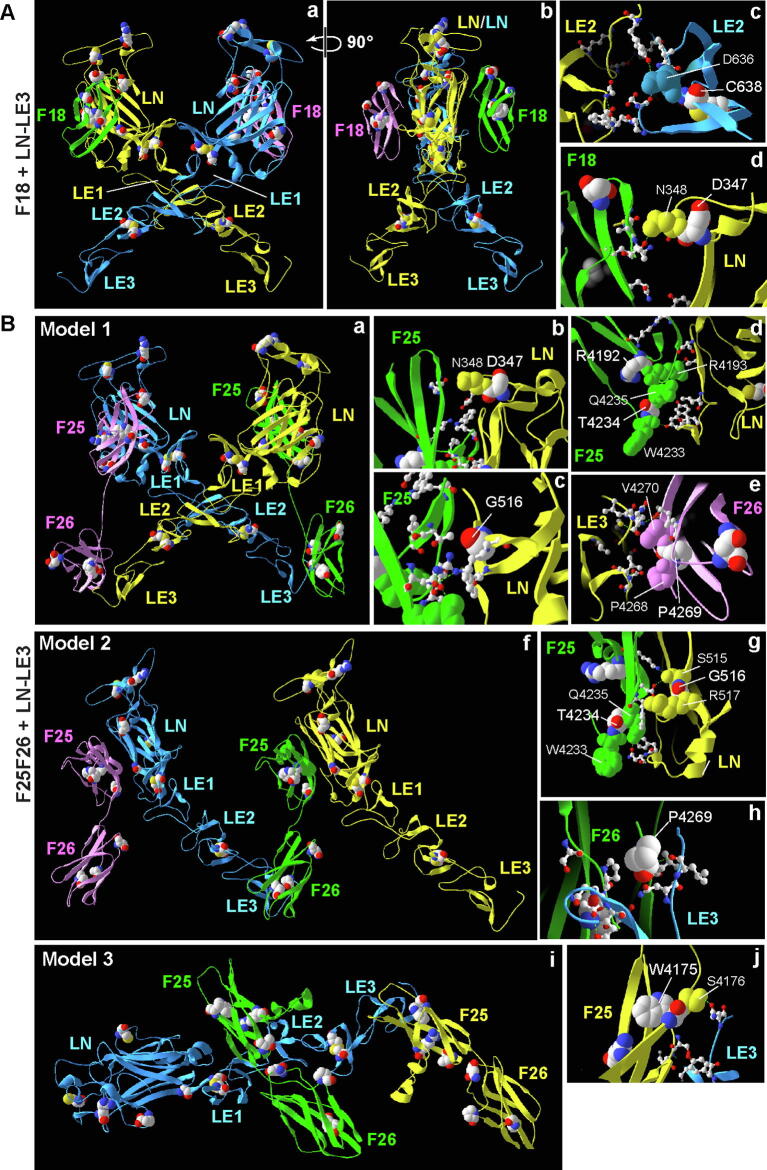
Usherin FN3 domains are predicted to interact with usherin LN and LE domains. (A) Two views of a 2:2 heterodimer model of usherin F18 and LN-LE3 fragments with a 90-degree rotation (a and b). C638 is next to D636 at the interface between the two LE2 domains (c). D347 in the LN domain is immediately next to N348 at the interface with the F18 domain (d). (B) Three models were generated based on templates of netrin-1 and its receptors (Table 3). Model 1 is similar to the model of the F18 and LN-LE3 complex. The F25 domain takes the place of the F18 domain, and the F26 domain associates with the LE3 domain (a). At the interface of F25 and LN domains, D347 is next to an interface residue N348 (b); G516 itself is an interface residue (c); R4192 is next to the interface residue R4193; and T4234 is next to the interface residues W4233 and Q4235 (d). At the interface between F26 and LE3 domains, P4269 is next to the interface residues P4268 and V4270 (e). Model 2 shows a tandem connection between usherin F25-F26 and LN-LE3 fragments, and the connection occurs at the interface between F25 and LN domains and the interface between F26 and LE3 domains (f). At the interface between F25 and LN domains, G516 is next to the interface residues S515 and R517. T4234 is next to the interface residues W4233 and Q4235 (g). At the interface between F26 and LE3 domains, P4269 is an interface residue (h). Model 3 shows a complex of one usherin LN-LE3 fragment and two usherin F25-F26 fragments. One F25-F26 fragment interacts with the LE1 and LE2 domains, and another F25-F26 fragment interacts with the LE3 domain (i). At the interface between F25 and LE3 domains, W4175 is next to the interface residue S4176 (j). The models are presented as ribbons and colored differently for protein fragments. The residues at the interface of two protein fragments are shown as a ball-stick model. Wild-type residues (CPK coloring), where missense mutations occur, and their neighboring interface residues (same color as protein fragments) are shown in a space-filling model. Green dashed lines indicate hydrogen bonds. (For interpretation of the references to color in this figure legend, the reader is referred to the web version of this article.)

The model of the usherin LN-LE3 and F18 complex was a 2:2 heterodimer, which was built on a template complex of netrin-1 LN-LE3 and neogenin F4-F5 fragments (Fig. 7Aa–b and Table 3). In this model, the usherin LN-LE3 fragment had a tadpole shape with the LN domain as the head and the three LE domains as the tail. The two LN-LE3 fragments crossed at the LE2 domain to form a symmetrical “X” shape. C638, the 8th cysteine in the LE2 domain, was next to the interface residue D636 (Fig. 7Ac), suggesting that the C638F mutation may disrupt the interaction between the two LE2 domains, in addition to its aforementioned potential disruption of the entire LE2 domain folding. Each of the two F18 domains interacted with one LN-LE3 fragment at the LN domain. The pathogenic missense mutation residue D347 in the LN domain was located next to interface residue N348 (Fig. 7Ad). Therefore, mutation D347H probably interferes with the interaction between usherin LN and F18 domains.

Three models of the usherin LN-LE3 and F25-F26 complex were generated based on complex templates of netrin-1 and its receptors. In the three models, the usherin LN-LE3 fragment adopted a tadpole conformation as in the model of the usherin LN-LE3 and F18 complex. In fact, model 1 was based on the same template as the usherin LN-LE3 and F18 complex (Fig. 7Ba and Table 3). Similarly, the two LN-LE3 fragments adopted a symmetrical “X” shape, and C638 was involved in the LE2-LE2 interaction. In this model, the F25 and F26 domains of two F25-F26 fragments interacted with the LN and LE3 domains on different LN-LE3 fragments, respectively. At the interface between LN and F25 domains, D347 was next to interface residue N348 (Fig. 7Bb); G516 itself was an interface residue (Fig. 7Bc); and R4192 and T4234 were next to interface residues R4193, W4233, and Q4235 (Fig. 7Bd). At the interface between F26 and LE3 domains, P4269 was next to interface residues P4268 and V4270 (Fig. 7Be).

Model 2 was based on a template of the complex between netrin-1 LN-LE3 and DDC F4-F5 fragments (Fig. 7Bf and Table 3). In this model, two LN-LE3 fragments were arranged in parallel and were connected by a F25-F26 fragment through an interaction between F25 and LN domains and an interaction between F26 and LE3 domains. The F25 domain of a second F25-F26 fragment associated with the spare LN domain of the two LN-LE3 fragments. At the interface between F25 and LN domains, G516 was next to interface residues S515 and R517, and T4234 was next to interface residues W4233 and Q4235 (Fig. 7Bg). At the interface between F26 and LE3 domains, P4269 itself was an interface residue (Fig. 7Bgh).

Model 3 was built on a crystal structure obtained from a complex of netrin-1 LN-LE3 and DDC F5-F6 fragments (Fig. 7Bi and Table 3). This model contained one LN-LE3 fragment and two F25-F26 fragments. The LN-LE3 fragment interacted with the F25 domain of one F25-F26 fragment through its LE3 domain and with the F25 and F26 domains of another F25-F26 fragment through its LE1 and LE2 domains. W4175 in F25 was next to residue S4176 at the interface with LE3 (Fig. 7Bj).

In summary, homology modeling based on complexes of netrin-1 LN-LE3 fragment with different netrin-1 receptor FN3 fragments suggests that usherin LN-LE3 and some FN3 domains are able to interact. Although the details of the interface residues are not exactly the same among the different complex models, D347H, G516V, C638F, R4192H, R4192C, T4234P, P4269R, and W4175G mutations are predicted to affect the interactions between usherin LN-LE3 and F18 or F25-F26 fragments.

### 3.7 Expression of full-length usherin and secretion of usherin ectodomain are low in mammalian cultured cells

**Fig. 8:**
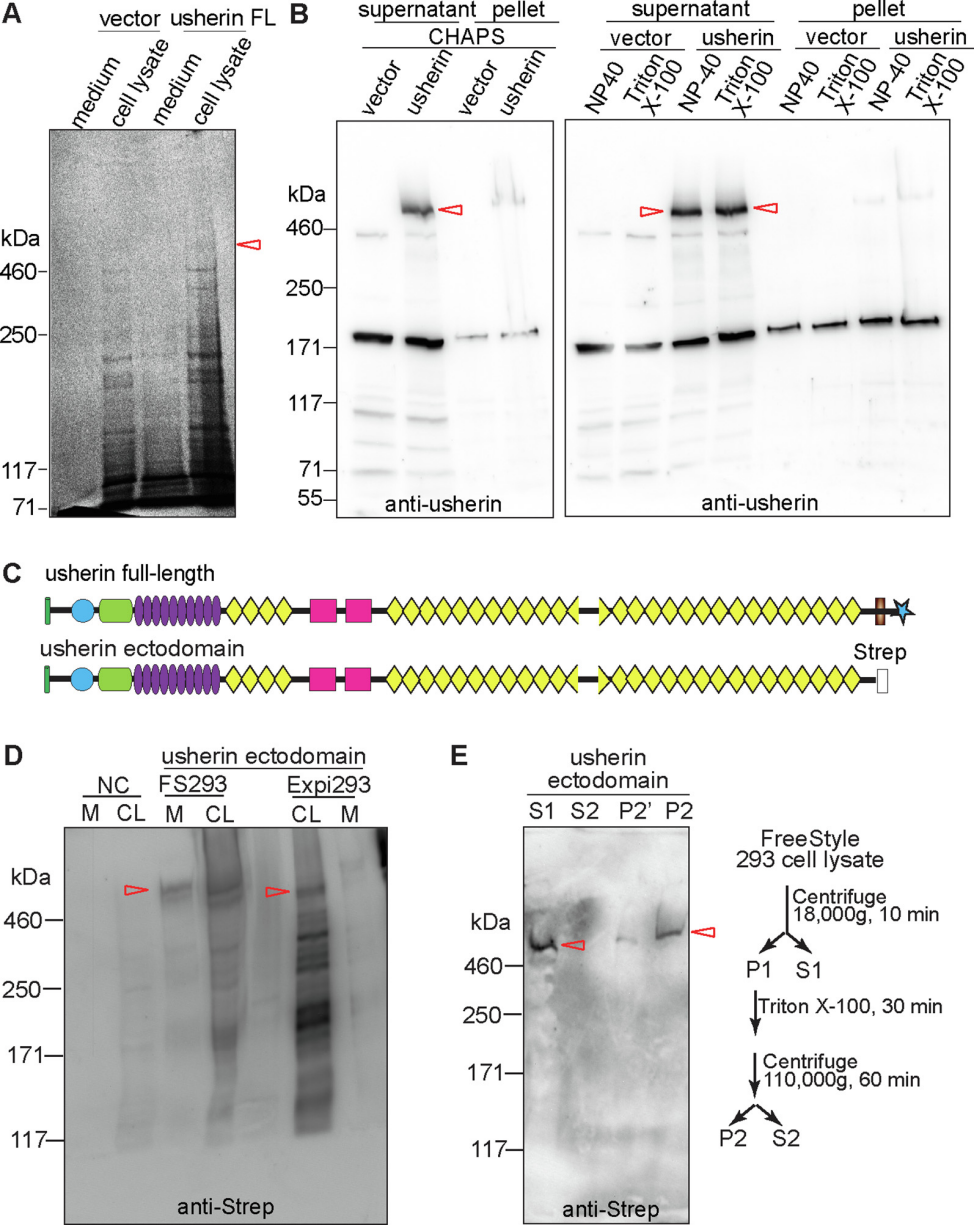
Expression of usherin and secretion of usherin ectodomain are low in mammalian cultured cells. (A) Coomassie blue-stained polyacrylamide gel shows that full-length (FL) usherin was expressed and remained in the cell lysate of transfected COS-7 cells. Note that the expression level of the recombinant usherin was barely detectable. (B) Immunoblotting analysis using usherin A3 antibody shows that the recombinant usherin, expressed in FreeStyle™ 293-F cells, was able to be solubilized by 0.5% CHAPS, 1% NP-40, and 1% Triton X-100. Note that the bands at ~180 kDa are non-specific. (C) A diagram of the Strep-tagged usherin ectodomain protein examined in this study. (D) Immunoblotting analysis using a Strep antibody shows that a small fraction of usherin ectodomain was secreted into the culture medium (M) when expressed in FreeStyle™ 293-F (FS293) cells, and little was secreted when expressed in Expi293F™ (Expi293) cells. CL, cell lysate; NC, non-transfected negative control cells. (E) Differential centrifugation and detergent extraction show that the majority of usherin ectodomain was localized in the cell cytosol (S1), and a small fraction was probably associated with cytoskeletons or cell membranes (P2). The procedures of differential centrifugation and detergent extraction are shown on the right. The P2′ lane is the same sample as the P2 lane, but the loading amount was one fourth of those of the S1, S2, and P2 lanes. Red arrows: the position of full-length usherin in A, the solubilized full-length usherin in B, and the expressed usherin ectodomain in D and E. (For interpretation of the references to color in this figure legend, the reader is referred to the web version of this article.)

It is crucial to express and purify high-quality usherin protein in a sufficient quantity in order to determine the usherin structure experimentally. To test this feasibility, we examined the expression of mouse full-length usherin with its own signal peptide in FreeStyle™ 293-F, Expi293F™, and COS-7 cells. Full-length usherin was expressed at a predicted molecular weight of ~570 kDa (Fig. 8A and B). As a transmembrane protein, usherin was not secreted into the culture medium (Fig. 8A). Its solubilization from cell membranes was achievable by incubation with zwitterionic or nonionic detergents, such as 0.5% CHAPS, 1% NP-40, or 1% Triton X-100 (Fig. 8B). However, the usherin protein expressed from ~10^6^ cells was barely detectable on a Coomassie blue-stained polyacrylamide gel (Fig. 8A), indicating that the protein yield was extremely low and insufficient for structural studies.

We then examined the expression of mouse usherin ectodomain with its endogenous signal peptide and a C-terminal Strep fusion (Fig. 8C) in FreeStyle™ 293-F and Expi293F™ cells. Immunoblotting analysis using an anti-Strep antibody showed that the majority of the expressed usherin ectodomain was not as expected to be secreted into the culture medium, especially when expressed in Expi293F™ cells (Fig. 8D), indicating that the usherin ectodomain may lose its native conformation and aggregate inside cells. Differential centrifugation and Triton X-100 extraction confirmed that most usherin ectodomain was present in the cytosol with a small fraction likely trapped with cell membranes or cytoskeletons (Fig. 8E).

### 3.8 Usherin FN3 fragments are expressed and secreted more robustly than usherin laminin-related fragments in mammalian cultured cells

**Fig. 9:**
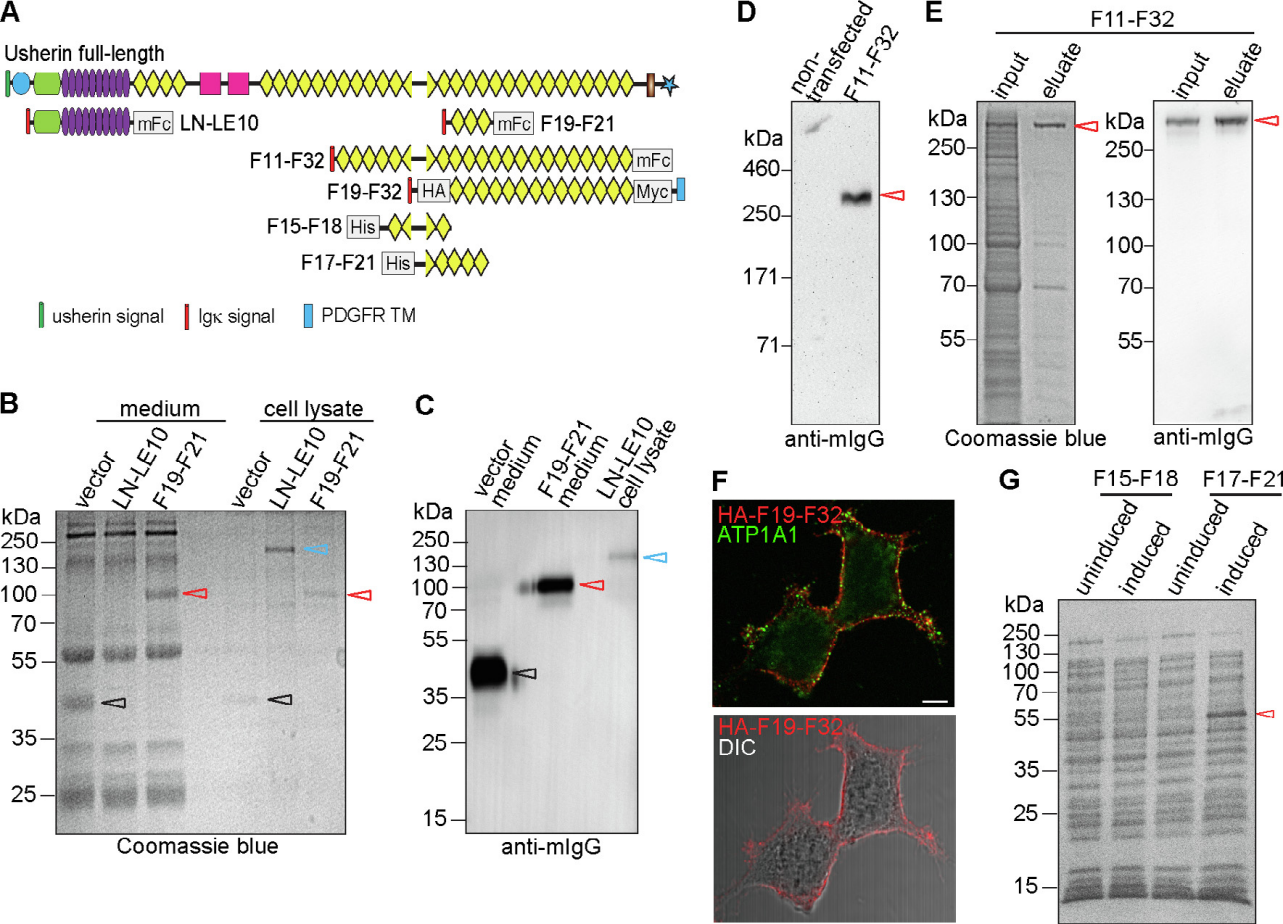
Usherin FN3 fragments are expressed and secreted more robustly than laminin-related fragments in cultured cells. (A) A scheme showing different usherin fragments examined in this study. (B) Coomassie blue-stained polyacrylamide gel shows that the mFc-tagged usherin F19-F21 fragment (red arrow), but not the mFc-tagged usherin LN-LE10 fragment (cyan arrow), was secreted into the culture medium when expressed in HEK293 cells. mFc expression (black arrow) from the empty vector was used as a control. (C) Immunoblotting analysis using an anti-mouse IgG antibody confirmed the bands shown on the polyacrylamide gel in B. (D) Immunoblotting analysis using an anti-mouse IgG antibody demonstrates the presence of the usherin F11-F32 fragment in the culture medium of transfected Expi293F™ cells. (E) Coomassie blue staining and immunoblotting analyses show successful affinity purification of the usherin F11-F32 fragment from culture medium using protein G resin. (F) Immunostaining demonstrates that the transfected usherin F19-F32 fragment was located on the plasma membrane, labeled by an antibody against ATP1A1, in COS-7 cells. Scale bar, 5 μm. (G) Coomassie blue-stained polyacrylamide gel shows expression of usherin F17-F21 fragment (red arrow) but little expression of usherin F15-F18 fragment, which includes the F16-F17 linker, in bacterial BL21 cells. (For interpretation of the references to color in this figure legend, the reader is referred to the web version of this article.)

The expression of mouse usherin LN-LE10 and F19-F21 fragments was investigated in HEK293 cells. The two usherin fragments were fused in-frame with a mouse Igκ signal peptide at their N-terminus and a mouse IgG2b Fc (mFc) and a biotinylation signal at their C-terminus (Fig. 9A). The recombinant LN-LE10 and F19-F21 proteins were expressed at the predicted molecular weights of ~130 kDa and ~95 kDa, respectively, where a chimera mFc-biotinylation signal protein was expressed at ~45 kDa (Fig. 9B). All these proteins were confirmed by immunoblotting analysis using an anti-mFc antibody (Fig. 9C). Compared with the usherin LN-LE10 fragment, the expression level and the secretion of the usherin F19-F21 fragment into the culture medium were more robust (Fig. 9B).

We then tested the expression of a large usherin FN3 fragment, F11-F32, which accounted for the C-terminal half of the usherin ectodomain and was fused with the mouse Igĸ signal peptide, mFc, and biotinylation signal (Fig. 9A). The F11-F32 fragment was detected in the culture medium of the transfected Expi293F™ cells at a molecular weight of ~340 kDa (Fig. 9D), which was slightly larger than the expected molecular weight (303 kDa) probably because of glycosylation. Through affinity purification using protein G, we were able to purify the F11-F32 fragment from the culture medium, as shown by the Coomassie blue-stained protein gel and immunoblotting analysis (Fig. 9E). The yield of the purified protein reached ~9 μg per 30 ml of culture medium, although a higher yield was still needed for structural studies.

We then tested another mammalian cell culture protein expression system to express usherin fragments. The usherin fragments were fused in-frame with an N-terminal BM40 (osteonectin, also known as SPARC) signal peptide and either a C-terminal human IgG2 Fc (hFc) fragment or a C-terminal FLAG tag. After expression in 293-EBNA cells, immunoblotting analysis using anti-hFc and/or anti-FLAG antibodies found that the usherin F5-F15, F17-F32, F17-F21, F17-F23, and F25-F32 fragments were expressed and secreted into the culture medium, while usherin F1-LG2 and F1-F15 fragments, which contained the LG1 and LG2 domains, were expressed at a lower level and remained in cell lysates (not shown). These findings were generally consistent with the results from the HEK293 and Expi293F™ cell protein expression systems using a DNA plasmid containing a mouse Igκ signal peptide, mFc tag, and biotinylation signal (Fig. 9B–E).

We further studied the subcellular localization of usherin F19-F32 fragment, which was fused with a mouse Igĸ signal peptide, a human platelet-derived growth factor receptor transmembrane domain, and an HA tag. After transfection in COS-7 cells, double immunostaining using antibodies against the HA tag and ATP1A1, a plasma membrane marker protein [50], [51], showed that the usherin F19-F32 fragment was localized at the plasma membrane, with no usherin aggregates observed in the cytoplasm (Fig. 9F). This result, together with the above observation that usherin FN3 fragments were secreted into the culture medium, indicated that the exogenously expressed usherin FN3 fragments are transported normally in mammalian cultured cells and thus probably fold in a native structural conformation.

We subsequently investigated whether usherin FN3 fragments and F16-F17 linker were able to be generated in a bacterial protein expression system, which is much more cost-effective than the mammalian cell culture system. The usherin F15-F18 and F17-F21 fragments were cloned and expressed in BL21 cells. In a Coomassie blue-stained polyacrylamide gel, the usherin F17-F21 fragment was detectable at a molecular weight of ~55 kDa, very close to its predicted size of 52.5 kDa, but the usherin F15-F18 fragment was not detectable (Fig. 9G). Therefore, the bacterial protein expression system can be used alternatively to express and produce short usherin FN3 fragments but not the F16-F17 linker.

## 4 Discussion

In this study, we built structural models for usherin LGL, LN, LE, LG, FN3, and transmembrane domains using homology modeling and for usherin F16-F17 linker and intracellular region using sequential sequence- and structure-based threading and *ab initio* modeling. Our studies show that usherin LGL, LN, LG, and FN3 domains adopt a β-sandwich conformation; LE repeat folding is maintained by disulfide bonds; transmembrane domain is a long α-helix; and both F16-F17 linker and intracellular region possess multiple loops among β-strands and α-helixes. Our structural models predict that some usherin FN3 domains interact among each other and with the usherin LN and LE domains. The usherin FN3 repeats are able to bend at the F16-F17 and F24-F25 linkers. Therefore, the usherin protein may have functionally relevant linear and bent conformations through multiple intramolecular and intermolecular interactions. Systematic investigation of 47 *USH2A* pathogenic small in-frame, mostly missense, mutations demonstrates that USH-associated mutations tend to be in the ultimate N-terminal domains, while RP-associated mutations tend to be in the ultimate C-terminal domains. Most of these mutations are localized at the periphery of the core β-sandwich domains, with some at or next to the interface with other domains, thereby probably affecting the protein function. Mutations in the LE region mostly occur at disulfide bonds. These mutations and the mutation in the transmembrane domain are predicted to affect the domain folding. Furthermore, our exploratory studies on the expression of usherin and its various fragments in mammalian cultured cells suggest that usherin FN3 fragments can be produced in their native conformation.

The templates used for homology modeling in this study share 16.5% to 30.4% sequence identity with various usherin fragments and complexes and cover the sequences of these fragments and complexes by 88% to 99% (Table 3), except for the template used for modeling the complex of F20-F23 and F25-F28 fragments (Table 3), which generated a dimer model only covering the F21-F23 and F25-F26 regions (Fig. 5C). Therefore, our models are expected to reasonably predict the overall fold of the secondary and tertiary structures, such as the position and topology of the β-strands in the β-sandwiches and the position and orientation of the disulfide bonds in the LE repeats. The F16-F17 linker and intracellular models generated by I-TASSER had a relatively low resolution because of the lack of homologous templates. Using these models, we were still able to localize the *USH2A* pathogenic missense mutations and roughly predict their effects on the usherin structure [45]. However, because the sequence identities of all our templates are below 40%, the models built in this study have a high error probability in the loops and residue side chains. We were thus unable to investigate exactly how the mutant residues change the structures in detail. For example, we cannot explain why the missense mutations at the same residue, R4192H and R4192C, cause different diseases, e.g., USH and RP, respectively. Additionally, some small in-frame variants may affect pre-RNA splicing instead of the encoded protein sequences, but this could occur to only a small fraction of the variants studied here and should not affect our main findings. In summary, under the current situation that the usherin structure has not been experimentally determined, our models provide a preliminary understanding of how usherin folds in order to function *in vivo* and how pathogenic small in-frame mutations affect the folding.

Our modeling suggests that usherin may form an antiparallel homodimer through interactions among multiple FN3 domains (Fig. 5A, B, and Table S3). The FN3 repeat region of the protein can extend linearly (Fig. 3B) or bend at multiple positions, such as at the F16-F17 and F24-F25 linkers (Fig. 6A and 3C, respectively). The interactions between the usherin LN/LE and FN3 domains (Fig. 7) may contribute to the antiparallel homodimer formation through intermolecular interactions or stabilize the bent conformation through intramolecular interactions. There are probably multiple structural conformations that usherin can adopt, which may determine usherin function under different physiological conditions, similar to what has been observed with the fibronectin protein [52]. The usherin missense mutations located directly at or immediately next to the interface between the interacting domains may cause diseases by disrupting the interactions (Fig. 5, Fig. 7). Additionally, tandem FN3 domains can be extended in response to mechanic stress [52]. Mutations at the interface of two adjacent FN3 domains, such as the T4234P and N4292D mutations (Fig. 3C), may affect the biophysical properties of the usherin protein. However, all the predictions from this modeling study are not sufficient to envision the actual structural conformation of full-length usherin *in vivo*, which needs significant improvement of homologous template structural data and determination of usherin and usherin fragment structures.

Usherin LE repeats were previously reported to interact with type IV collagen and fibronectin [12], [13]. It was shown that mutations R535T in loop b of LE1, G713R in loop b of LE4, and C536R in LE1 affect the interaction with type IV collagen, while mutations L555V in loop d of LE1, C572S in LE1, and C620F in LE 2 affect the interaction with fibronectin [12], [13]. Our modeling shows that cysteines in the LE repeats are essential for protein folding and that G713 faces outward in loop b of LE4 (Fig. 2Da). We thus conclude that both loop b of LE1 and LE4 are involved in type IV collagen binding and that loop d of LE1 and some region of LE2 are involved in fibronectin binding. Our modeling also shows that D778 in loop d of LE5 faces outward, suggesting that mutation D778Y in LE5 may affect binding with an unknown partner. Furthermore, C759F mutation in LE5 disrupts a disulfide bond and probably interfere with another neighboring disulfide bond, thereby causing a protein folding defect and likely compromising the protein function. Finally, both homology modeling (SWISS MODEL) and iterative threading assembly refinement modeling (I-TASSER) predict that exon 13 skipping disrupts usherin folding between LE4 and LE8 (Fig. 2D). This structural defect is presumably similar to those caused by missense mutations at a single cysteine in LE5 (C759F) and LE8 (C934W) and the D778Y mutation, which are mostly associated with RP (Table 1 and [10]), indicating that the usherin LE4-LE8 region is important for a photoreceptor-specific function. Therefore, exon 13 skipping may not rescue retinal degeneration caused by small in-frame mutations in exon 13, but this strategy may be able to partially rescue retinal degeneration caused by truncating mutations in exon 13, such as the c.2299delG mutation. Additionally, the folding in other regions of the usherin ΔEx13 protein, especially the remaining LE4 part and the neighboring LE3 and LE9 domains, appeared normal (Fig. 2D). Thus, usherin ΔEx13 protein may still function, which explains the normal hearing function in *Ush2a*^ΔEx12^ mice [32], [33].

The differential localization of USH- and RP-associated homozygous mutations in usherin domains could be explained by two nonexclusive possibilities. First, the *USH2A* gene has been proposed to encode two alternative splicing protein isoforms, a long isoform, which was studied here, and a short N-terminal isoform, which terminates after the F4 domain [53]. Although the short isoform was not detected in the retina [18], it may exist in the inner ear and play a unique function in addition to the long isoform. Second, the partners that interact with the N- and C-terminal usherin domains may be different in photoreceptors and hair cells. For example, PDZD7 interacts with the C-terminal usherin PBM in hair cells but not in photoreceptors [14]. In this study, further investigation found no correlation of phenotypes with missense mutations in terms of their changes in residue charge, size, and hydrophobicity; their positions in β-strand, α-helix, and loop structures; and their locations relative to the periphery and core of the tertiary structures. Therefore, it is necessary to generate high-resolution structural models or to experimentally determine the actual structures of usherin to gain more insight into the *USH2A* genotype-phenotype correlation and to explain why different mutations in the same domains, different mutations at the same residues, and the same missense mutations lead to different disease phenotypes.

Our studies on usherin protein expression *in vitro*, especially in mammalian cultured cells, demonstrate that a significant effort is required to optimize the culture conditions in order to finally achieve large-scale production of usherin full-length and ectodomain proteins for structural investigation. At this time, the production of usherin FN3 fragments appears to be more feasible than the production of usherin laminin-related fragments. Alternatively, expression of short usherin FN3 fragments in the traditional and cost-effective bacterial cell culture system may be considered.

In summary, we have generated structural models for various domains of usherin, a major causative protein in inherited retinal degeneration and sensorineural hearing loss, using homology modeling and sequence- and structure-based threading with *ab initio* building. These models provide us with novel insights into the usherin structure, intramolecular and/or intermolecular interactions, and the pathogenic mechanisms of *USH2A* small in-frame mutations. Although more work is needed to overcome the technical difficulties in usherin protein production and structure determination, our current results serve as a foundation for future hypothesis formation and experimental assessment. Our findings of the differential distribution of USH- and RP-associated small in-frame mutations and the effect of exon 13 deletion on LE repeats will improve our understanding of the *USH2A* genotype-phenotype correlation and inform future therapeutic development.

## 5 Data availability statement

All data are described in the manuscript, in addition to supplemental Tables S1–S3.

## CRediT authorship contribution statement

**Dongmei Yu:** Conceptualization, Investigation, Methodology, Funding acquisition. **Junhuang Zou:** Investigation. **Qian Chen:** Investigation. **Tian Zhu:** Investigation. **Ruifang Sui:** Investigation, Supervision. **Jun Yang:** Conceptualization, Investigation, Methodology, Writing - original draft, Funding acquisition, Writing - review & editing, Supervision.

## Declaration of Competing Interest

The authors declare that they have no known competing financial interests or personal relationships that could have appeared to influence the work reported in this paper.
