# Supplementary material for: Structural modeling, mutation analysis, and in vitro expression of usherin, a major protein in inherited retinal degeneration and hearing loss
Source: Comput Struct Biotechnol J. 2020 Jun 10;18:1363–82. doi: 10.1016/j.csbj.2020.05.025 (PMC7317166; doi:10.1016/j.csbj.2020.05.025)
Supplement: Supplementary data 1 [file mmc1.docx]

**Table S1**. Usherin FN3 domain alignment among different species.

| New # ^1^ | **Human**  NP_996816 | | **Mouse**  NP_067383 | | **Rat**  NP_001289148 | | **Chicken**  XP_015139380 | | **Zebrafish**  XP_009291422 | |
| --- | --- | --- | --- | --- | --- | --- | --- | --- | --- | --- |
|  | # ^2^ | Range (aa) | # ^2^ | Range (aa) | # ^2^ | Range (aa) | # ^2^ | Range (aa) | # ^2^ | Range (aa) |
| **F1** | F1 | 1060-1143 | F1 | 1055-1128 | F1 | 1054-1127 | F1 | 1071-1156 |  | 1074-1157 ^3^ |
| **F2** | F2 | 1145-1238 | F2 | 1153-1235 | F2 | 1152-1234 | F2 | 1170-1251 | F1 | 1177-1253 |
| **F3** | F3 | 1242-1357 | F3 | 1239-1351 | F3 | 1238-1350 | F3 | 1255-1371 | F2 | 1264-1375 |
| **F4** | F4 | 1368-1451 | F4 | 1362-1445 | F4 | 1361-1444 | F4 | 1380-1461 | F3 | 1386-1469 |
| **F5** | F5 | 1956-2051 | F5 | 1947-2042 | F5 | 1946-2041 | F5 | 1965-2062 | F4 | 1981-2071 |
| **F6** |  | 2054-2135 ^4^ |  | 2045-2126 ^4^ | F6 | 2044-2125 | F6 | 2065-2149 |  | 2074-2154 ^4^ |
| **F7** |  | 2143-2238 ^5^ | F6 | 2134-2230 | F7 | 2133-2223 | F7 | 2154-2247 | F5 | 2163-2253 |
| **F8** | F6 | 2241-2313 | F7 | 2232-2304 | F8 | 2231-2303 | F8 | 2252-2324 | F6 | 2258-2341 |
| **F9** | F7 | 2329-2428 | F8 | 2320-2419 | F9 | 2319-2418 | F9 | 2340-2440 | F7 | 2352-2443 |
| **F10** | F8 | 2432-2528 | F9 | 2423-2516 | F10 | 2422-2518 | F10 | 2443-2539 | F8 | 2447-2544 |
| **F11** | F9 | 2533-2619 | F10 | 2524-2610 | F11 | 2530-2608 | F11 | 2554-2630 | F9 | 2547-2633 |
| **F12** | F10 | 2621-2719 | F11 | 2612-2710 | F12 | 2610-2708 | F12 | 2632-2730 | F10 | 2635-2733 |
| **F13** | F11 | 2724-2799 | F12 | 2715-2807 | F13 | 2713-2788 | F13 | 2732-2830 | F11 | 2743-2807 |
| **F14** | F12 | 2818-2920 | F13 | 2809-2911 | F14 | 2810-2909 | F14 | 2832-2931 | F12 | 2830-2932 |
| **F15** | F13 | 2925-3015 | F14 | 2916-2993 | F15 | 2914-3004 | F15 | 2936-3026 | F13 | 2939-3014 |
| **F16** | F14 | 3026-3087 |  | 3017-3078 ^3^ |  | 3015-3076 ^3^ | F16 | 3037-3102 | F14 | 3032-3099 |
| **F17** | F15 | 3449-3494 | F15 | 3441-3486 | F16 | 3439-3484 | F17 | 3461-3506 |  | 3474-3519 ^3^ |
| **F18** | F16 | 3503-3586 |  | 3497-3578 ^3^ | F17 | 3487-3564 | F18 | 3515-3598 | F15 | 3542-3611 |
| **F19** | F17 | 3590-3676 | F16 | 3582-3668 | F18 | 3584-3662 | F19 | 3602-3688 | F16 | 3615-3701 |
| **F20** | F18 | 3680-3767 | F17 | 3672-3753 | F19 | 3670-3748 | F20 | 3698-3779 | F17 | 3718-3784 |
| **F21** | F19 | 3777-3856 | F18 | 3769-3854 | F20 | 3762-3852 | F21 | 3782-3865 | F18 | 3801-3887 |
| **F22** | F20 | 3866-3960 | F19 | 3867-3952 | F21 | 3865-3950 | F22 | 3885-3972 | F19 | 3908-3971 |
| **F23** | F21 | 3964-4057 | F20 | 3956-4053 | F22 | 3954-4051 | F23 | 3983-4073 | F20 | 3996-4086 |
| **F24** |  | 4066-4150 ^6^ |  | 4058-4142 ^6^ |  | 4056-4140 ^6^ | F24 | 4078-4162 |  | 4091-4175 ^6^ |
| **F25** | F22 | 4161-4247 | F21 | 4153-4239 | F23 | 4151-4248 | F25 | 4173-4264 | F21 | 4186-4284 |
| **F26** | F23 | 4268-4351 | F22 | 4260-4343 | F24 | 4258-4341 | F26 | 4280-4363 | F22 | 4288-4366 |
| **F27** | F24 | 4356-4439 | F23 | 4348-4431 | F25 | 4346-4418 | F27 | 4368-4451 | F23 | 4382-4465 |
| **F28** | F25 | 4444-4516 | F24 | 4435-4519 | F26 | 4433-4517 | F28 | 4455-4539 | F24 | 4470-4553 |
| **F29** | F26 | 4529-4627 | F25 | 4521-4619 | F27 | 4519-4617 | F29 | 4541-4639 | F25 | 4555-4653 |
| **F30** | F27 | 4636-4730 | F26 | 4645-4722 | F28 | 4643-4710 | F30 | 4669-4742 | F26 | 4679-4753 |
| **F31** |  | 4732-4809 **^e^** | F27 | 4724-4800 | F29 | 4722-4798 | F31 | 4744-4821 | F27 | 4760-4830 |
| **F32** | F28 | 4826-4927 | F28 | 4817-4918 | F30 | 4815-4916 | F32 | 4849-4942 | F28 | 4864-4955 |

^1^ FN3 domain numbers annotated in this study.

^2^ FN3 domain numbers annotated in the NCBI conserved domain database.

^3^ Determined by BLASTp using human sequence.

^4^ Determined by BLASTp using rat sequence.

^5^ Determined by BLASTp using mouse sequence.

^6^ Determined by BLASTp using chicken sequence.

Highlighted FN3 domains are partial FN3.

**Table S2**. Sequence identity of usherin and FN3 domains between human and other species.

| **FN3 #/Usherin** | **Mouse** | **Rat** | **Chicken** | **Fish** |
| --- | --- | --- | --- | --- |
| F1 | 75.0% | 79.2% | 60.7% | 46.4% |
| F2 | 68.7% | 71.1% | 64.6% | 40.8% |
| F3 | 61.9% | 61.9% | 66.7% | 50.9% |
| F4 | 57.1% | 60.7% | 60.7% | 44.1% |
| F5 | 67.0% | 73.2% | 74.2% | 59.1% |
| F6 | 62.2% | 65.9% | 64.2% | 55.6% |
| F7 | 63.5% | 67.0% | 62.8% | 47.9% |
| F8 | 82.2% | 80.8% | 80.8% | 60.3% |
| F9 | 61.0% | 62.0% | 53.0% | 37.4% |
| F10 | 78.7% | 77.3% | 68.0% | 54.6% |
| F11 | 69.0% | 67.1% | 55.8% | 48.3% |
| F12 | 72.7% | 73.7% | 74.8% | 46.5% |
| F13 | 72.4% | 77.6% | 60.5% | 52.2% |
| F14 | 86.4% | 84.0% | 77.0% | 60.2% |
| F15 | 75.6% | 72.5% | 57.1% | 44.7% |
| F16 | 87.1% | 79.0% | 90.3% | 51.6% |
| F16-F17 linker | 71.6% | 68.5% | 63.3% | 46.2% |
| F17 | 65.2% | 56.5% | 63.0% | 50.0% |
| F18 | 68.3% | 65.7% | 71.4% | 60.9% |
| F19 | 72.4% | 77.9% | 66.7% | 56.3% |
| F20 | 75.6% | 76.0% | 61.3% | 53.0% |
| F21 | 70.9% | 65.8% | 57.9% | 50.0% |
| F22 | 73.3% | 75.6% | 68.2% | 67.2% |
| F23 | 70.2% | 73.4% | 65.5% | 59.8% |
| F24 | 83.5% | 81.2% | 75.3% | 69.4% |
| F25 | 63.2% | 66.7% | 65.5% | 40.9% |
| F26 | 65.5% | 63.1% | 62.7% | 42.5% |
| F27 | 68.1% | 64.4% | 54.8% | 41.6% |
| F28 | 80.8% | 79.5% | 74.0% | 63.0% |
| F29 | 65.7% | 67.7% | 53.5% | 51.5% |
| F30 | 71.8% | 70.6% | 64.9% | 54.9% |
| F31 | 69.2% | 74.4% | 70.5% | 58.3% |
| F32 | 65.7% | 69.6% | 53.2% | 45.1% |
| Entire Protein | 71.3% | 71.5% | 66.3% | 51.9% |

FN3 domains highlighted in red are evolutionarily more conserved as shown in Fig. 4B.

FN3 domains highlighted in grey are evolutionarily less conserved as shown in Fig. 4B.

Mouse, rat, chicken, and fish FN3 domains highlighted in yellow are less featured as shown in Fig. 1A.

**Table S3**. Quality scores for the homodimer models of 4 consecutive usherin FN3 domains based on template 3t1w.1.A.

| **Fragment** | **Range (aa)** | **Seq identity** | **Coverage** | **GMQE** | **QMEAN** | **QSQE** |
| --- | --- | --- | --- | --- | --- | --- |
| F1-F4 | 1060-1451 | 19.5% | 86% | 0.53 | -4.68 | 0.05 |
| F13-F16 | 2724-3087 | 19.9% | 90% | 0.56 | -4.36 | 0.15 |
| F20-F23 | 3680-4057 | 18.5% | 90% | 0.56 | -3.98 | 0.12 |
| F25-F28 | 4161-4516 | 18.9% | 91% | 0.57 | -3.88 | 0.15 |
| F28-F31 | 4444-4809 | 20.3% | 92% | 0.57 | -3.80 | 0.16 |
